# Supplementary material for: Mutations observed in somatic evolution reveal underlying gene mechanisms
Source: Commun Biol. 2023 Jul 19;6:753. doi: 10.1038/s42003-023-05136-y (PMC10356810; doi:10.1038/s42003-023-05136-y)
Supplement: Supplementary file 2 — Supplementary Information [file 42003_2023_5136_MOESM2_ESM.pdf]

Supplementary Information

This supplementary text discusses our statistical technique in relation to cancer driver detection methods that are based on the same underlying principle (section 1). We also explore the assumptions of the statistical method using the NOTCH1 ligand-binding region (sections 2–4). We also discuss several factors may influence the distribution of mutations across NOTCH1 (section 5), and analyse the patterns of missense mutations in *NOTCH2*, *TP53* and *PIK3CA* (sections 6-8).

Supplementary Note 1. Related work

There are a number of methods that use a similar statistical principle to detect genes under selection. They assume that mutations are generated according to the mutational spectrum (Figure S1, methods 5.7-5.8). In genes where no mutations of any kind convey a growth advantage/disadvantage, the mutations detected by DNA sequencing will simply be an unbiased sample of the mutations produced by the spectrum. In genes under selection, it is assumed that certain types of mutations (missense/nonsense mutations [1], or mutations estimated by machine-learning approaches to have high functional impact [2,3]) are more likely to alter cell phenotype than others (silent mutations, mutations with low estimated functional impact). Therefore, more high-impact mutations will be detected in a positively selected gene than would be expected under the neutral null hypothesis [1-3] (Figure S1). Conversely, negative selection in a gene will lead to the detection of fewer high-impact mutations than would be expected under the null hypothesis.

Our analysis method is based on the same principles as the cancer driver detection tools (Figure S1). However, it differs from the driver detection tools in two key ways. First, the method is set up to be flexible - allowing the selection of many different aspects of mutations to be tested within the same framework. Secondly, and crucially, the method is able to distinguish and separately test selection due to different functional changes within the same region – as multiple forms of selection can obscure each other if not properly accounted for (Figure 1).

These differences mean that our tool performs a different function from the driver detection methods. These driver detection methods are designed to return a comprehensive-as-possible

set of genes under selection, and to do so use scores that represent the overall severity of the functional impact of mutations. Our tool, in contrast, does not come with a default scoring method – you can test for any functional impact that can be represented as a numerical score for each mutation. Testing for a single functional impact will not detect drivers that are under selection for a different mutational effect, and testing the same dataset with multiple scores risks producing false positives.

Our method is best suited to detailed investigation of individual genes. Unlike the driver detection tools, our method can link selection with particular alterations to gene mechanisms and can separate multiple types of selected mutation in the same region. The tool can be used in combination with driver detection tools, which can narrow down candidate genes for detailed investigation, and prior knowledge can be incorporated to tailor the statistical analysis to the gene of interest.

Supplementary Note 2. Choice of mutational spectrum assumptions

The form of the mutational spectrum must be defined to construct the null model (Figure S1, methods 5.7-5.8). In this section we explore a range of spectrum assumptions and their impact on the statistical tests of selection in *NOTCH1* EGF11–12.

The probability of a particular somatic mutation appearing in a sequenced sample depends on both the rate at which the mutation occurs in cells and the strength of selection on the mutation once it has occurred. The statistical method attempts to separate these two factors, and therefore requires a model of how often each mutation would appear in the absence of selection (the null hypothesis model). A common method to do this is to assume that, within a gene, each mutation in the same spectrum category has the same mutation rate [1-3]. For example, using a trinucleotide spectrum, two AAA trinucleotide sequences at different locations within the same gene are assumed to have the same probability of mutating into ACA.

A variety of spectrum assumptions have been used in driver detection models. Using a trinucleotide mutational spectrum to generate a null model has been found to improve driver detection compared to more simplistic substitution models [1]. However, it has also been suggested that a pentanucleotide context may be more appropriate for the signature of ultraviolet light [1]. Transcription coupled repair may produce a strand bias in the mutational

spectrum [1] which can be taken into account by separating the transcribed and non-transcribed strand of a gene. Previous studies have assumed either that all coding regions in the genome share the same mutational spectrum [1,2], or that each gene has its own spectrum [3]. Null models may improve in the future as work continues to determine the factors that influence mutation rate [4].

Each of these assumptions alters the number of mutation rate parameters that need to be estimated. The more mutation rate parameters there are, the more thinly spread the observed mutations, and the noisier the estimation of each rate will become. The choice of spectrum assumptions is therefore a compromise between a potentially more accurate representation of the context that determines mutation rate (larger spectrum) and more stable estimates of each rate parameter (smaller spectrum).

Selected mutations within the data may distort the mutational spectrum. This could be a larger problem when using gene specific spectra or for datasets containing only a small number of genes because the selected mutations may make up a larger proportion of the total data. To some extent this selection bias can be reduced by removing duplicate mutations before calculating the spectrum [5]. For high mutation loads, where duplicate mutations are likely even under neutral selection, this deduplication may have its own distorting effect.

We ran the tests for selection of destabilising, ligand interface, and calcium-binding mutations in *NOTCH1* EGF11–12 (section **3.1.1 in the main text**) using a range of different spectrum assumptions to see how robust the signs of selection are to a variety of null hypotheses. In all cases, the results remained significant (Figure S4, Table S2). The least significant results tended to be from spectra with large numbers of mutational categories calculated from only the mutations in *NOTCH1* (Figure S4, Table S2). These are likely to be examples of overly detailed spectra for the data [3], with up to 3072 separate rate parameters, calculated in the case of the oesophagus from less than 1300 single nucleotide substitutions in *NOTCH1*. As the number of mutations per rate parameter reduces, recurrent hotspots will have a larger influence over their own expected mutation rate in the null model. In the extreme case, where each possible mutation has a unique rate parameter, the expected frequencies in the null model would simply match the observed data.

Supplementary Note 3. Both selection and mutation rate contribute to the mutations observed

Testing selection in *NOTCH1* EGF11–12 using a range of different mutation rate assumptions found that the statistical results were not substantially altered and the inference of selection was robust (Supplementary Note 2 above). However, this does not mean the mutation spectrum is irrelevant, and the transmembrane region of *NOTCH1* provides an illustration of the importance of considering the mutational spectrum when analysing the observed patterns of mutation.

The NOTCH1 intracellular domain (NICD1) is released by cleavage of the transmembrane helix of NOTCH1 at the S3 site by the multiprotein γ-secretase complex [6] (Figure 2). The exact site of cleavage can vary, but the S3-V cleavage (between G1753 and V1754) produces the most stable form of NICD1 due to N-end rule degradation [7] and is therefore likely responsible for the majority of *NOTCH1* signalling [8]. Mutations in the vicinity of the S3 cleavage site may affect *NOTCH1* signalling by reducing S3 cleavage efficiency [9] or by leading to the production of less stable NICD1 species [7].

There are far fewer mutations in the transmembrane region than the EGF repeats of *NOTCH1* (Figure 3a), and the majority of missense mutations occur on a single residue (Figure S5a,b). In this case, we could not look for common selected features since it lacked the required diversity of different mutations (Supplementary Note 4 below). The hotspot residue, G1753, is on a highly conserved residue adjacent to the S3 cleavage site at which γ-secretase cleaves the NICD from the extracellular domain of NOTCH1 [10] (Figure S5a,b). Preventing cleavage or shifting the cleavage site to produce an NICD with a shorter half-life would inactivate, or at least reduce, the *NOTCH1* signal [7-9].

Conservation of protein sequence across species is likely to be correlated with function, with residues that are more important for normal protein function more likely to be conserved [11-14]. G1753 is highly conserved, but so are other residues in the transmembrane region that were not mutated in the skin or oesophagus datasets (Figure S5a,b). This possibly suggests that mutations on these non-mutated residues were not selected as strongly as G1753R/E, despite the high conservation scores. However, looking at both the conservation score and the mutational spectrum, we see that G1753R and G1753E are the only mutations that are both on

highly conserved nucleotides and that have high expected mutation rates (Figure S5c,d), plausibly explaining why it was these mutations in particular that appeared recurrently in the data sets.

This shows the importance of considering the mutational spectrum when investigating which mutations are selected for. Within *NOTCH1* EGF11–12, unsurprisingly, mutations with a higher expected mutation rate were generally more mutated (Figure S6). However, low sample size, imprecise mutation rate estimation, and potential within-category differences in functional impact mean that it is hard to precisely predict which individual mutations will be highly mutated (Figure S6). This illustrates one advantage of testing for mutation function selection using the bulk set of mutations instead of focusing on individual hotspot mutations. Supplementary Text Section 4 discusses another advantage of using the bulk set of mutations: that hotspots can lead to false inference of functional selection.

Supplementary Note 4. Correlation, causation and hotspots

The statistical method tests for a shift in the distribution of a metric between the null model and the observed mutations (Figure S1). Ideally, any shift detected will be due to selection of the particular feature that is being tested. However, hotspots can distort the distribution, regardless of the metric used, and can even provide evidence of selection when using random numbers to score the mutations [3]. This is not necessarily a problem when just looking for evidence of selection acting on a gene. However, the aim of this work is to infer whether the particular feature is selected, and distortions due to hotspots may give a false impression of the importance of a feature (Figure S7a,b)

A simple approach is to check that the trend of selection is still apparent after deduplicating the recurrent mutations (Figure S7c,d). P-values from this approach may not be reliable because hotspots may be expected to occur if the feature is strongly selected, and deduplicating mutations both reduces the sample size and could distort the mutational spectrum used for the null model (see section 2 above). However, it can still be a useful method to explore the influence of hotspots.

We used two null models: one with a spectrum calculated from the full data, as used elsewhere in this study; the other with a spectrum calculated using deduplicated data (section 2 above).

The trends of selection in *NOTCH1* EGF11–12 for destabilising mutations, ligand-binding interface mutations and calcium-binding mutations are still apparent and statistically significant after the removal of duplicate mutations (Figure S7d-l). This provides more confidence that the significant results obtained for *NOTCH1* EGF11–12 are due to the selection of the features identified.

Supplementary Note 5. Multiple hypotheses could explain the distribution of mutations across *NOTCH1*

So far we have only looked at small regions of *NOTCH1*. More information is potentially available from the distribution of mutations across the whole gene. For example, the ligand-binding EGF repeats were much more mutated than the rest of *NOTCH1* (Figure S3a). There are a number of possible explanations for this, which are not mutually exclusive.

Firstly, it could be a result of a variable background mutation rate across the gene; that is to say, some regions of the gene are inherently more prone to mutation. Synonymous mutations have been used to estimate background mutation rates [15] as they are assumed to be neutrally selected. There is a significantly higher number of synonymous mutations in EGF11–12 than the rest of the gene (Figure S8a,b, skin: expected=3%, observed=9%, $p=5.6e^{-9}$, n=404; oesophagus: expected=3%, observed=19%, p=0.0005, n=31; two-tailed binomial test, Supplementary Methods), suggesting that a variable mutation rate could be contributing to the uneven distribution of missense mutations. However, some synonymous mutations may have been created in the same mutation event as a nearby missense mutation and not successfully merged into a single multi-nucleotide variant [16], meaning the synonymous mutation is carried along as a passenger. It is also possible that some of the synonymous mutations are not functionally neutral [17] and therefore may be increased or decreased in number due to positive or negative selection, meaning they may not give a true indication of mutation rate.

Secondly, it may be that the DNA sequence of the highly mutated regions has a higher probability of acquiring mutations due to the mutational spectrum. However, this does not appear to be the case for missense mutations (Figure S8c,d). Thirdly, it could be that a higher proportion of potential mutations are functionally impactful in the ligand-binding region than other sections of the gene. For example, we have seen that approximately two-thirds of potential single nucleotide substitutions in the EGF11–12 repeats are likely to be selected. If

this proportion is lower in the rest of the gene, this would result in a smaller number of observed mutations there.

Fourthly, the effects of a disruptive mutation may be milder in the non-ligand-binding regions. It might be that disruption of ligand binding completely stops the *NOTCH1* signal, but, for example, a mutation in the transmembrane helix reduces rather than entirely stops the signal [7]. Lastly, it has been suggested that NOTCH receptors may form homodimers or clusters when they bind to a ligand [18,19]. Potentially, this could mean that a mutant in the ligand-binding region could disrupt the activity of the wild type allele as well as the mutant allele, reducing the *NOTCH1* signal more than having completely lost one *NOTCH1* allele (Figure S8e). This dominant negative effect has been observed for some missense mutations in the *TP53* DNA-binding domain [20]. The last two hypotheses suggest that the fitness of *NOTCH1* mutant clones depends on the dose of *NOTCH1* signalling. This is consistent with the frequent observation of loss-of-heterozygosity copy number variants associated with *NOTCH1* mutations [21], which strongly suggests that losing both wild type alleles of *NOTCH1* provides a stronger growth advantage than losing a single allele. This *Notch1* dose-dependent fitness was also confirmed to be the case in lineage tracing studies of mice [22].

Supplementary Note 6. *NOTCH2* EGF11–12 displays similar patterns of selection to *NOTCH1*

NOTCH1 and *NOTCH2* are highly similar genes, with the same mechanism of activation (Figure 2). Both are drivers of clonal expansion in skin and oesophagus and both have a peak of missense mutations at the ligand-binding EGF repeats 11 and 12 (Figure S11a) [13,16,21]. Therefore, based on the results for *NOTCH1*, we considered destabilising, ligand-binding interface and calcium-binding mutations as potentially selected groups in *NOTCH2* EGF11–12 (Figure S11b,c; total missense mutations in region: skin 178, oesophagus 38). To remove confounding sources of selection, we tested each category while excluding mutations in the other two categories from both the null and observed distributions. Destabilising mutations (excluding ligand-binding and calcium-binding mutations; skin: $p<2e^{-5}$, n=98; oesophagus: $p<2e^{-5}$, n=25; two-tailed Monte Carlo test, Supplementary Methods, Figure S11d,e), ligand-interface mutations (excluding $\Delta\Delta G>2$kcal/mol and calcium-binding mutations; skin: expected=27%, observed=56%, $p=2e^{-5}$, n=48; oesophagus: expected=23%, observed=67%, p=0.03, n=6; two-tailed binomial test, Supplementary Methods, Figure S11f,g), and calcium-

binding mutations (excluding $\Delta\Delta G > 2$kcal/mol and ligand-binding mutations; skin: expected=14%, observed=34%, p=0.003, n=32; oesophagus: expected=15%, observed=60%, p=0.03, n=5; two-tailed binomial test, Supplementary Methods, Figure S11h,i) were significantly enriched compared to the null models. The results show that selection of missense mutations in the *NOTCH2* ligand-binding EGF repeats follows similar patterns to selection in *NOTCH1* in both skin and oesophagus.

Supplementary Note 7. Missense mutations in *TP53* destabilise the protein and disrupt DNA binding

Along with *NOTCH1*, *TP53* is one of the two most significantly selected genes in both the oesophagus and skin [16,21]. Truncating mutations in *TP53* are strongly selected [16,21] and most missense mutations in *TP53* occur in the DNA-binding domain (total missense mutations in DNA-binding domain: skin 898, oesophagus 380; Figure S12a). We therefore hypothesised that, similar to *NOTCH1* and *NOTCH2*, the missense mutations in the DNA-binding domain are inactivating *TP53* by either causing protein misfolding or by affecting the interaction surface of p53 (in this case the binding interface with DNA). We found strong enrichment of destabilising mutations (excluding mutations within 5Å of the DNA molecule; skin: $p<2e^{-5}$, n=760; oesophagus: $p<2e^{-5}$, n=338; two-tailed Monte Carlo test, Supplementary Methods, Figure S12b,c,f) and of mutations close to the DNA molecule (excluding mutations with $\Delta\Delta G>2$ kcal/mol; skin: $p<2e^{-5}$, n=395; oesophagus: $p<2e^{-5}$, n=154; two-tailed Monte Carlo test, Supplementary Methods, Figure S12d,e,f). Approximately 42% of potential mutations in the *TP53* DNA-binding domain are either destabilising ($\Delta\Delta G>2$ kcal/mol) or within 5Å of the bound DNA molecule, and together these two categories contain 71% and 69% of observed mutations in skin and oesophagus respectively.

Supplementary Note 8. Positive and negative selection in PIK3CA

The lipid-kinase PI3K is made up of the subunits p110α, encoded by *PIK3CA*, and p85α, which binds to p110α to inhibit PI3K activity [23]. In the normal skin, dN/dS analysis found missense mutations were under approximately neutral selection, while nonsense and splice mutations are negatively selected [16] (Figure S7). In contrast, missense mutations in the normal oesophagus are positively selected, with recurrent mutations of the H1047R hotspot [21] (Figure S13a).

We compared the observed missense mutations to those catalogued in Clinvar [24], a curated database of disease associated genetic mutations. In both skin and oesophagus, we found a significant enrichment of mutations matching those labelled in Clinvar as pathogenic/likely pathogenic (skin: expected=2%, observed=12%, $p=4e^{-9}$, n=167; oesophagus: expected=2%, observed=45%, $p=3e^{-25}$, n=49; two-tailed binomial test, Supplementary Methods, Figure S13b,c). In particular, the matching mutations were associated with various cancers and overgrowth diseases known to be driven by *PIK3CA* activating mutations.

To further investigate the missense mutations, we looked for enrichment of mutations in key regions of the protein that undergo conformational changes when PI3K is activated by a phosphopeptide or binds to a membrane [25] (Table S1). These regions include the locations of many cancer-linked *PIK3CA* mutations such as G106V, G118D, N345K, E542K, E545K and H1047R. In skin there is enrichment in residues 100–119, 444–473, and 962–980 (Figure S13d-f,i, Table S3). Although there are many recurrent mutations in the gene (Figure S13a), these trends are still apparent when removing duplicate mutations (Table S4, Supplementary Text section 3), with only the 444–473 region not quite reaching statistical significance. Residues 100–119 are part of the link between the adapter-binding domain (ABD) and the Ras-binding domain (RBD). Mutations in this region have been found to cause conformational changes normally associated with membrane binding and to increase kinase activity [25]. Residues 444–473 are on the interface between p110α and p85α (Figure S13i), the disruption of which can lead to activation of PI3K [25]. Residues 962–980 are in the kinase domain and are involved with membrane binding [25,26]. Together with the enrichment of mutations in Clinvar, these analyses provide strong evidence that, despite the apparently neutral results of dN/dS analysis, selection is acting on *PIK3CA* missense mutations in normal skin. The analyses suggest that there is negative selection of inactivating mutations (shown by the dN/dS ratio of truncating mutations of less than one, Figure S9) and positive selection of activating mutations. The combination of these opposing directions of selection may contribute to the inference of neutral selection when missense mutations in *PIK3CA* are analysed as a whole [1].

In oesophagus there is enrichment of missense mutations in residues 100–119 (also enriched in skin) and 1039–1055 (Figure S13ag-i, Table S3). Residues 1039–1055 are at the C-terminal end of the kinase domain and contain the strongly activating H1047R mutation [25]. Despite the

high frequency of hotspots – the H1047R hotspot in particular – both regions remain significantly selected after removing duplicate mutations (Table S4).

Supplementary Note 9. Guide to running analyses

A more comprehensive guide to the method is available here: https://github.com/michaelhall28/darwinian_shift/wiki.

This guide explains how to run basic and more complex analysis and includes numerous Python code examples.

Supplementary Note 10. Supplementary Methods

10.1 Protein structures

The following structures were used for the individual protein analysis of FoldX ΔΔG and structural distance calculations:

NOTCH1 EGF11–12	2VJ3 [27]
NOTCH2 EGF11–12	5MWB [13]
TP53 DNA-binding domain	2AC0 chain A [28]
PIK3CA	4L1B [29]
FBXW7	2OVQ [30]

Residue numbers in the PDB files were aligned to residue numbers in the UniProt protein sequences using SIFTS [31]. Distances between residues in structures were calculated using the Python package MDAnalysis [32].

For the comparison of nonsense dN/dS values and FoldX ΔΔG, UniProt [33] was used to find all protein structures for genes sequenced in skin that had resolution ≤ 2.6Å, no mutations in the protein sequence, and containing at least 50 missense mutations in the skin data set. To reduce computational expense, only a single chain (the first alphabetically for the protein of interest) was run per protein structure. The structures run were:

TP53	1TSR [34], 1TUP [34], 1YCS [35], 2AC0 [28], 2ADY [28], 2AHI [28], 2ATA [28], 2OCJ [36], 2XWR [37], 2YBG [38], 3IGL [39], 3KMD [40], 3KZ8 [39], 4HJE [41], 4QO1 [42], 4XR8 [43], 5BUA [44], 5MCT [45], 5MCU [45], 5MCV [45], 5MCW [45], 5MF7 [45], 5MG7 [45], 6FJ5 [45]
NOTCH1	1YYH [46], 2F8Y [47], 2VJ3 [27], 3ETO [48], 3L95 [49], 4CUD [50], 4D0E [50], 5FMA [51], 5L0R [52]
GRIN2A	5H8F [53], 5H8H [53], 5H8N [53], 5H8Q [53], 5I2N [54], 5KCJ [53], 5KDT [54], 5TP9 [55], 5TPA [55]
PIK3CA	4L1B [29], 4L23 [29], 5DXT [56], 5UBR [57], 5XGI [58], 6GVF [59], 6PYS [60]
ERBB4	2R4B [61], 3BCE [62], 3U2P [63]
FBXW7	2OVQ [30], 2OVR [30], 5V4B [64]
ERBB2	1N8Z [65], 2A91 [66], 5MY6 [67]
EGFR	1MOX [68], 1YY9 [69], 4UV7 [70]
TP63	3QYN [71], 3US0 [72]
NOTCH3	4ZLP [73], 5CZX [74]
EPHA2	3FL7 [75]
BAI3	4DLO [76]
PTPRT	2OOQ [77]
NOTCH2	5MWB [13]
GRM3	3SM9 [78]

10.2 Homology models of mouse proteins

Where no experimental structure could be found for a gene in Uniprot using the primary accession, SwissModel was used to find an homology structure. The ddg calculations were used for sections from the homology structure that exactly matched the gene residues. To find homology structures the primary accession for the gene was sourced from Uniprot, and used to call the API: https://swissmodel.expasy.org/repository/uniprot/{accession}.json

gene	homology model	accession	residues
abcb11	afq9qy30-f1-model_v3	Q9QY30	1-1321
abcb11	smhom_6lr0_1_a_44_1315	Q9QY30	44-1315
abcb11	smhom_6s7p_1_a_47_1295	Q9QY30	47-1295
abcb11	smhom_6c0v_1_a_46_1319	Q9QY30	46-1319
abcc2	afq8vi47-f1-model_v3	Q8VI47	1-1543
abcc2	smhom_6uy0_1_a_188_1536	Q8VI47	188-1536
abcc2	smhom_5uj9_1_a_188_1522	Q8VI47	188-1522
abcc2	smhom_7mpe_1_a_20_1530	Q8VI47	20-1530

abcc2	smhom_7s61_1_e_3_1526	Q8VI47	3-1526
abcc2	smhom_7m69_1_a_145_1509	Q8VI47	145-1509
abcc2	smhom_6c3o_1_h_3_1530	Q8VI47	3-1530
adam10	afo35598-f1-model_v3	O35598	1-749
adam10	smhom_6be6_4_a_219_655	O35598	219-655
adam29	afq811q4-f1-model_v3	Q811Q4	1-763
adam29	smhom_5y31_1_a_201_665	Q811Q4	201-665
adam29	smhom_2ero_1_a_198_626	Q811Q4	198-626
adam29	smhom_3k7n_1_a_200_625	Q811Q4	200-625
adam29	smhom_6be6_1_a_203_513	Q811Q4	203-513
adam29	smhom_3b2z_1_a_202_505	Q811Q4	202-505
adcy10	afq8c0t9-f1-model_v3	Q8C0T9	1-1614
adcy10	smhom_4oyp_1_a_1_467	Q8C0T9	1-467
aff3	afp51827-f1-model_v3	P51827	1-1254
aff3	smhom_6r80_1_a_992_1253	P51827	992-1253
aff3	smhom_4ogr_3_c_47_111	P51827	47-111
akt1	afp31750-f1-model_v3	P31750	1-480
akt1	smhom_3d0e_1_a_145_478	P31750	145-478
akt1	smhom_6hhg_1_a_3_444	P31750	3-444
akt1	smhom_4y93_1_a_4_405	P31750	4-405
akt2	afq60823-f1-model_v3	Q60823	1-481
akt2	smhom_3d0e_1_a_146_480	Q60823	146-480
akt2	smhom_6hhg_1_a_3_445	Q60823	3-445
akt2	smhom_4y93_1_a_4_406	Q60823	4-406
arid1a	afa2bh40-f1-model_v3	A2BH40	1-2283
arid1a	smhom_1ryu_1_a_1001_1119	A2BH40	1001-1119
arid2	afe9q7e2-f1-model_v3	E9Q7E2	1-1828
arid2	smhom_7vdv_1_m_16_522	E9Q7E2	16-522
arid5b	afq8bm75-f1-model_v3	Q8BM75	1-1188
arid5b	smhom_1ig6_1_a_320_425	Q8BM75	320-425
arid5b	smhom_5k4l_1_a_257_414	Q8BM75	257-414
asxl1	afp59598-f1-model_v3	P59598	1-1514
asxl1	smhom_6hgc_1_b_249_345	P59598	249-345
atm	smhom_7sic_1_a_5_3066	Q62388	5-3066
atm	smhom_6k9l_1_a_2_3064	Q62388	2-3064
atp2a2	afo55143-f1-model_v3	O55143	1-1044
atp2a2	smhom_6lle_1_a_1_1044	O55143	1-1044
atp2a2	smhom_6lly_1_a_1_1044	O55143	1-1044
atrx	afq61687-f1-model_v3	Q61687	1-2476
atrx	smhom_1z3i_1_a_1534_2199	Q61687	1534-2199
bbs9	afq811g0-f1-model_v3	Q811G0	1-885
bbs9	smhom_6vbv_1_h_2_827	Q811G0	2-827
bbs9	smhom_6xt9_1_d_9_799	Q811G0	9-799
bcl11b	afq99pv8-f1-model_v3	Q99PV8	1-884
bcl11b	smhom_6ki6_1_a_784_868	Q99PV8	784-868
bcl11b	smhom_2wbs_1_a_785_866	Q99PV8	785-866
bcl11b	smhom_5und_1_a_783_870	Q99PV8	783-870
bcr	afq6paj1-f1-model_v3	Q6PAJ1	1-1270

bcr	smhom_4yon_1_a_490_867	Q6PAJ1	490-867
bcr	smhom_5fi0_2_a_490_867	Q6PAJ1	490-867
bcr	smhom_3jzy_1_a_909_1005	Q6PAJ1	909-1005
bcr	smhom_1kzg_1_a_491_868	Q6PAJ1	491-868
bcr	smhom_3ksy_1_a_492_867	Q6PAJ1	492-867
braf	afp28028-f1-model_v3	P28028	1-751
braf	smhom_7mfd_1_a_140_723	P28028	140-723
braf	smhom_7z37_1_c_499_708	P28028	499-708
braf	smhom_5vyk_1_a_59_121	P28028	59-121
c1s	afq8cg14-f1-model_v3	Q8CG14	1-688
c1s	smhom_6f1c_1_d_17_292	Q8CG14	17-85
c1s	smhom_4lmf_6_b_18_290	Q8CG14	18-85
c1s	smhom_6f1c_1_c_16_291	Q8CG14	16-85
c1s	smhom_4j1y_1_a_294_684	Q8CG14	314-381
cacna1d	afq99246-f1-model_v3	Q99246	1-2179
cacna1d	smhom_7uhg_1_b_121_1609	Q99246	121-1609
cacna1d	smhom_6jp8_1_d_107_1609	Q99246	107-1609
casp8	afo89110-f1-model_v3	O89110	1-480
casp8	smhom_4jj7_1_a_225_479	O89110	225-479
casp8	smhom_7lvj_1_a_1_185	O89110	1-185
casp8	smhom_5h31_1_a_2_183	O89110	2-183
casp8	smhom_5h33_1_a_2_182	O89110	2-182
ccnd1	afp25322-f1-model_v3	P25322	1-295
ccnd1	smhom_2w96_1_a_6_265	P25322	6-265
ccnd1	smhom_7nvs_1_m_55_260	P25322	55-260
ccnd1	smhom_5wh1_3_a_56_261	P25322	56-261
ccnd1	smhom_5wh1_2_a_56_261	P25322	56-261
ccnd1	smhom_1tfb_1_a_55_262	P25322	55-262
cdc16	afq8r349-f1-model_v3	Q8R349	1-620
cdc16	smhom_6q6g_1_i_2_566	Q8R349	2-566
cdc16	smhom_5lcw_1_c_5_525	Q8R349	5-525
chuk	afq60680-f1-model_v3	Q60680	1-745
chuk	smhom_5ebz_1_a_15_660	Q60680	15-660
clgn	afp52194-f1-model_v3	P52194	1-611
clgn	smhom_1jhn_1_a_51_446	P52194	51-446
clgn	smhom_7qpd_1_e_60_453	P52194	60-453
clgn	smhom_1jhn_1_a_280_399	P52194	280-399
clgn	smhom_3rg0_1_a_60_444	P52194	60-444
cnot1	afq6zq08-f1-model_v3	Q6ZQ08	1-2375
cnot1	smhom_4j8s_1_a_819_1000	Q6ZQ08	819-1000
cnot1	smhom_4gml_3_a_1092_1313	Q6ZQ08	1092-1313
cnot1	smhom_4c0d_1_a_1841_2352	Q6ZQ08	1841-2352
cnot1	smhom_8bfi_1_a_1_661	Q6ZQ08	1-661
cntnap4	afq99p47-f1-model_v3	Q99P47	1-1310
cntnap4	smhom_5mc9_1_a_182_556	Q99P47	182-556
cntnap4	smhom_6cw1_1_a_181_533	Q99P47	181-533
cntnap4	smhom_3asi_1_a_794_1183	Q99P47	794-1183
cntnap4	smhom_2wjs_1_a_183_550	Q99P47	183-550

cntnap4	smhom_6cw1_1_a_797_1178	Q99P47	797-1178
cntnap4	smhom_3r05_1_a_183_1206	Q99P47	183-1206
cntnap4	smhom_3qcw_1_a_183_1201	Q99P47	183-1201
cobll1	afq3umf0-f1-model_v3	Q3UMF0	1-1273
cobll1	smhom_6jh1_1_d_102_256	Q3UMF0	102-256
cobll1	smhom_3rt3_1_a_102_256	Q3UMF0	102-256
cobll1	smhom_5h07_1_a_100_256	Q3UMF0	100-256
col12a1	smhom_1fnf_1_a_1385_1727	Q60847	1385-1727
col12a1	smhom_1fnf_1_a_722_1078	Q60847	722-1078
col12a1	smhom_1fnf_1_a_1754_2109	Q60847	1754-2109
col12a1	smhom_1fnf_1_a_813_1170	Q60847	813-1170
col12a1	smhom_3t1w_1_a_631_991	Q60847	631-991
col12a1	smhom_3t1w_1_a_813_1176	Q60847	813-1176
col12a1	smhom_3t1w_1_a_1845_2205	Q60847	1845-2205
col12a1	smhom_3t1w_1_a_1385_1744	Q60847	1385-1744
col12a1	smhom_7nwl_1_c_631_986	Q60847	631-986
col12a1	smhom_3t1w_1_a_1754_2116	Q60847	1754-2116
col12a1	smhom_4cn9_2_a_133_622	Q60847	133-622
col12a1	smhom_4yh7_1_a_629_903	Q60847	629-903
col12a1	smhom_7nwl_1_c_1658_2014	Q60847	1658-2014
col12a1	smhom_6tpw_1_a_1655_2025	Q60847	1655-2025
col12a1	smhom_3t1w_1_a_726_1085	Q60847	726-1085
col12a1	smhom_6mfa_1_a_1655_2017	Q60847	1655-2017
cr2	afp19070-f1-model_v3	P19070	1-1025
cr2	smhom_2xrd_1_a_145_398	P19070	145-398
cr2	smhom_3iyp_1_e_80_336	P19070	80-336
cr2	smhom_6v09_1_a_140_463	P19070	140-463
ctcf	afq61164-f1-model_v3	Q61164	1-736
ctcf	smhom_5und_1_a_348_518	Q61164	348-518
ctcf	smhom_5v3g_1_c_263_430	Q61164	263-430
ctcf	smhom_5v3m_1_c_261_518	Q61164	261-518
ctcf	smhom_5und_1_a_263_430	Q61164	263-430
ctcf	smhom_5und_1_a_262_402	Q61164	262-402
ctcf	smhom_5v3m_1_c_265_546	Q61164	265-546
ctcf	smhom_5wjq_1_c_261_518	Q61164	261-518
ctcf	smhom_5wjq_1_c_263_578	Q61164	263-578
ctcf	smhom_5und_1_a_266_402	Q61164	266-402
ctcf	smhom_5v3m_1_c_321_591	Q61164	321-591
ctcf	smhom_5v3j_2_c_265_579	Q61164	265-579
cyld	afq80tq2-f1-model_v3	Q80TQ2	1-952
cyld	smhom_1whm_1_a_228_306	Q80TQ2	228-306
cyld	smhom_2vhf_2_a_579_951	Q80TQ2	579-951
cyld	smhom_3wxg_1_a_579_951	Q80TQ2	579-951
cyld	smhom_1whl_1_a_125_206	Q80TQ2	125-206
cyld	smhom_1ixd_1_a_456_546	Q80TQ2	456-546
dclk1	afq9jlm8-f1-model_v3	Q9JLM8	1-756
dclk1	smhom_6kyq_1_a_396_717	Q9JLM8	396-717
dclk1	smhom_4atu_1_i_55_160	Q9JLM8	55-160

dclre1a	afq9jic3-f1-model_v3	Q9JIC3	1-1026
dclre1a	smhom_4b87_1_a_683_1026	Q9JIC3	683-1026
ddr2	afq62371-f1-model_v3	Q62371	1-854
ddr2	smhom_4ag4_1_a_29_367	Q62371	29-367
ddr2	smhom_6y23_1_a_526_850	Q62371	526-850
dnm2	afp39054-f1-model_v3	P39054	1-870
dnm2	smhom_5wp9_1_a_6_493	P39054	6-493
dnm2	smhom_5a3f_1_a_6_739	P39054	6-739
dnm2	smhom_7ax3_1_a_3_742	P39054	3-741
dnm2	smhom_4bej_2_a_6_740	P39054	6-739
dnm2	smhom_6s9a_1_a_665_737	P39054	665-737
egfr	afq01279-f1-model_v3	Q01279	1-1210
egfr	smhom_7syd_1_c_25_638	Q01279	25-638
egfr	smhom_3bbt_1_a_704_994	Q01279	704-994
egfr	smhom_5o7p_1_a_28_636	Q01279	28-636
eif2d	afq61211-f1-model_v3	Q61211	1-570
eif2d	smhom_5oa3_1_a_1_568	Q61211	1-568
eif2d	smhom_6vpr_2_a_473_563	Q61211	473-563
eif2d	smhom_7trc_1_b_92_182	Q61211	92-182
ep300	afb2rws6-f1-model_v3	B2RWS6	1-2412
ep300	smhom_5hp0_1_a_1727_1818	B2RWS6	1727-1818
ep300	smhom_6gyr_2_a_1045_1663	B2RWS6	1045-1663
ep300	smhom_1p4q_1_b_324_424	B2RWS6	324-424
erbb2	afp70424-f1-model_v3	P70424	1-1256
erbb2	smhom_1iij_1_a_649_678	P70424	649-678
erbb2	smhom_6bgt_1_a_23_630	P70424	23-630
erbb2	smhom_3bbt_1_a_711_1000	P70424	711-1000
erbb3	afq61526-f1-model_v3	Q61526	1-1339
erbb3	smhom_7mn8_1_a_28_630	Q61526	28-630
erbb3	smhom_5o7p_1_a_26_629	Q61526	26-629
erbb3	smhom_3cs9_1_a_695_969	Q61526	695-969
erbb4	afq61527-f1-model_v3	Q61527	1-1308
erbb4	smhom_3u7u_2_a_26_637	Q61527	26-637
erbb4	smhom_2ahx_1_a_26_641	Q61527	26-641
erbb4	smhom_3bbt_1_a_708_998	Q61527	708-998
erbb4	smhom_3u9u_4_a_27_636	Q61527	27-636
fat4	smhom_6vg4_1_a_898_1505	Q2PZL6	898-1505
fat4	smhom_6e6b_1_a_49_681	Q2PZL6	49-681
fbn2	smhom_5ms9_1_a_143_317	Q61555	143-317
fbn2	smhom_1uzj_3_a_1524_1684	Q61555	1524-1684
fbxw7	afq8vbv4-f1-model_v3	Q8VBV4	1-710
fbxw7	smhom_5v4b_1_b_266_709	Q8VBV4	266-709
fbxw7	smhom_2ymu_1_a_368_706	Q8VBV4	368-706
fbxw7	smhom_6wnx_2_a_279_657	Q8VBV4	279-657
fbxw7	smhom_6jlq_1_b_376_707	Q8VBV4	376-707
fbxw7	smhom_5ylz_1_m_325_623	Q8VBV4	325-623
fgfr3	afq61851-f1-model_v3	Q61851	1-801
fgfr3	smhom_5a46_1_a_452_754	Q61851	452-754

fgfr3	smhom_1ry7_1_b_144_355	Q61851	144-355
flg2	afq2vis4-f1-model_v3	Q2VIS4	1-2362
flg2	smhom_4pcw_1_a_2_89	Q2VIS4	2-88
flt3	afq00342-f1-model_v3	Q00342	1-1000
flt3	smhom_7qdp_1_g_165_532	Q00342	165-532
flt3	smhom_7qdp_1_f_75_527	Q00342	75-527
flt3	smhom_6il3_1_a_570_950	Q00342	570-950
grin2a	afp35436-f1-model_v3	P35436	1-1464
grin2a	smhom_4tll_1_b_32_842	P35436	32-842
grin2a	smhom_4tll_1_d_32_842	P35436	32-842
grin2a	smhom_6mml_1_d_34_837	P35436	34-837
grin2a	smhom_6whs_1_b_33_844	P35436	33-844
grm3	afq9qys2-f1-model_v3	Q9QYS2	1-879
grm3	smhom_7mts_1_a_30_839	Q9QYS2	30-839
grm3	smhom_7mtq_1_a_31_828	Q9QYS2	31-827
gtf3c5	afq8r2t8-f1-model_v3	Q8R2T8	1-520
gtf3c5	smhom_4bji_1_a_215_465	Q8R2T8	215-465
hist1h2bm	afp10854-f1-model_v3	P10854	1-126
hist1h2bm	smhom_6m2m_1_d_37_124	P10854	37-124
hist1h2bm	smhom_3x1v_1_f_28_126	P10854	28-126
huwe1	smhom_7mop_1_a_17_4367	Q7TMY8	17-4367
huwe1	smhom_7n6g_17_a_299_593	Q7TMY8	299-593
huwe1	smhom_6s9o_1_a_388_634	Q7TMY8	388-634
hydin	smhom_7sqc_127_a_235_606	Q80W93	235-606
hydin	smhom_7n6g_31_a_89_468	Q80W93	89-468
igsf1	afq7tqa1-f1-model_v3	Q7TQA1	1-1317
igsf1	smhom_6grt_2_a_569_1139	Q7TQA1	569-1139
igsf1	smhom_6grs_1_a_570_1137	Q7TQA1	573-1135
igsf1	smhom_6grs_1_a_664_1229	Q7TQA1	667-1227
igsf1	smhom_6grt_1_a_664_1230	Q7TQA1	664-1230
insrr	afq9wtl4-f1-model_v3	Q9WTL4	1-1300
insrr	smhom_5u8q_1_a_28_909	Q9WTL4	28-909
insrr	smhom_7sl3_1_a_23_912	Q9WTL4	23-912
insrr	smhom_7sl1_1_a_23_911	Q9WTL4	23-911
insrr	smhom_7v3p_1_a_28_909	Q9WTL4	28-909
insrr	smhom_7sl2_1_a_25_909	Q9WTL4	25-909
insrr	smhom_7sti_1_a_25_909	Q9WTL4	25-909
insrr	smhom_6jk8_1_b_28_912	Q9WTL4	28-912
insrr	smhom_6jk8_1_a_28_916	Q9WTL4	28-916
iqgap1	afq9jkf1-f1-model_v3	Q9JKF1	1-1657
iqgap1	smhom_5l0o_1_a_28_191	Q9JKF1	28-191
iqgap1	smhom_3fay_1_a_962_1339	Q9JKF1	962-1339
iqgap1	smhom_5cjp_1_f_962_1333	Q9JKF1	962-1333
iqgap1	smhom_4eza_2_a_1559_1652	Q9JKF1	1559-1652
iqgap1	smhom_6sl7_1_a_50_201	Q9JKF1	50-201
iqgap1	smhom_7sqc_52_a_748_913	Q9JKF1	748-913
irf6	afp97431-f1-model_v3	P97431	1-467
irf6	smhom_3dsh_1_a_214_445	P97431	214-445

irf6	smhom_5jek_2_a_221_450	P97431	221-450
irf6	smhom_2o6g_1_e_5_116	P97431	5-116
kcnh5	afq920e3-f1-model_v3	Q920E3	1-988
kcnh5	smhom_5k7l_1_a_8_718	Q920E3	8-718
kdm6a	afo70546-f1-model_v3	O70546	1-1401
kdm6a	smhom_3avr_1_a_886_1395	O70546	886-1395
kdm6a	smhom_7y4i_1_b_74_427	O70546	74-427
kdm6a	smhom_4buj_1_b_77_423	O70546	77-423
kdm6a	smhom_6af0_1_a_73_427	O70546	73-427
kdm6a	smhom_8bbe_1_b_73_426	O70546	73-426
kdm6a	smhom_7kw7_1_e_170_432	O70546	170-432
kdm6a	smhom_7dkh_1_a_75_426	O70546	75-426
kdr	afp35918-f1-model_v3	P35918	1-1345
kdr	smhom_3vhk_1_a_812_1166	P35918	812-1166
kdr	smhom_3v2a_1_a_134_331	P35918	134-331
klrc3	afq9qxn7-f1-model_v3	Q9QXN7	1-246
klrc3	smhom_3cdg_1_d_101_218	Q9QXN7	146-166
kmt2c	smhom_4ynp_2_a_4752_4902	Q8BRH4	4752-4902
kmt2d	smhom_5v9j_1_b_5435_5586	Q6PDK2	5435-5586
kras	afp32883-f1-model_v3	P32883	1-189
kras	smhom_4dst_1_a_2_181	P32883	2-181
krt5	afq922u2-f1-model_v3	Q922U2	1-580
krt5	smhom_7d9n_1_b_161_313	Q922U2	161-313
krt5	smhom_6jlb_1_c_158_370	Q922U2	158-370
krt5	smhom_7rro_153_a_165_314	Q922U2	165-314
krt5	smhom_7uti_1_q_165_441	Q922U2	165-441
krt5	smhom_7uti_1_r_165_441	Q922U2	165-441
krt5	smhom_7utl_1_s_213_469	Q922U2	213-469
loxhd1	afc8yr32-f1-model_v3	C8YR32	1-2068
loxhd1	smhom_2fnq_1_a_972_1077	C8YR32	972-1077
loxhd1	smhom_2fnq_1_a_553_662	C8YR32	553-662
loxhd1	smhom_3vf1_1_a_172_275	C8YR32	172-275
loxhd1	smhom_3cwz_1_b_811_937	C8YR32	811-937
loxhd1	smhom_3vf1_1_a_43_148	C8YR32	43-148
loxhd1	smhom_3cwz_1_b_1947_2067	C8YR32	1947-2067
loxhd1	smhom_3cwz_1_b_1251_1375	C8YR32	1251-1375
lrp1	smhom_4dg6_1_a_200_846	Q91ZX7	200-846
lrp1	smhom_3s2k_1_a_1267_1887	Q91ZX7	1267-1887
lrp1	smhom_3m0c_1_c_1146_1581	Q91ZX7	1146-1581
lrp1	smhom_5gje_1_a_1585_2195	Q91ZX7	1585-2195
lrp1	smhom_3m0c_1_c_2905_3333	Q91ZX7	2905-3333
lrp1	smhom_3m0c_1_c_3742_4178	Q91ZX7	3742-4178
lrp1	smhom_3s94_1_a_200_845	Q91ZX7	200-845
lrp1	smhom_3m0c_1_c_1104_1580	Q91ZX7	1104-1580
lrp1	smhom_1n7d_1_a_2691_3334	Q91ZX7	2691-3334
lrp1	smhom_1n7d_1_a_3534_4184	Q91ZX7	3534-4184
lrp1b	smhom_3s94_1_a_1574_2185	Q9JI18	1574-2185
lrp1b	smhom_4dg6_1_a_1575_2190	Q9JI18	1575-2190

lrp1b	smhom_3m0c_1_c_1136_1571	Q9JI18	1136-1571
lrp1b	smhom_4a0p_1_a_1259_1875	Q9JI18	1259-1875
lrp1b	smhom_4dg6_1_a_203_833	Q9JI18	203-833
lrp1b	smhom_3m0c_1_c_2890_3315	Q9JI18	2890-3315
lrp1b	smhom_3m0c_1_c_3758_4202	Q9JI18	3758-4202
lrp2	smhom_4dg6_1_a_393_1014	A2ARV4	393-1014
lrp2	smhom_5gje_1_a_1438_2059	A2ARV4	1438-2059
lrp2	smhom_3m0c_1_c_1312_1743	A2ARV4	1312-1743
lrp2	smhom_6l6r_1_a_2064_2695	A2ARV4	2064-2695
lrp2	smhom_3m0c_1_c_265_705	A2ARV4	265-705
lrp2	smhom_4dg6_1_a_1747_2392	A2ARV4	1747-2392
lrp2	smhom_3m0c_1_c_3070_3511	A2ARV4	3070-3511
lrp2	smhom_3m0c_1_c_3922_4358	A2ARV4	3922-4358
lrp2	smhom_1n7d_1_a_2862_3512	A2ARV4	2862-3512
lrp2	smhom_1n7d_1_a_3718_4365	A2ARV4	3718-4365
ltf	afp08071-f1-model_v3	P08071	1-707
ltf	smhom_2bjj_1_a_20_706	P08071	20-706
ltf	smhom_1dtz_1_a_19_707	P08071	19-707
maml1	afq6t264-f1-model_v3	Q6T264	1-1020
maml1	smhom_3v79_1_c_16_70	Q6T264	16-70
maml1	smhom_6e1j_1_a_250_280	Q6T264	250-280
mcm7	afq61881-f1-model_v3	Q61881	1-719
mcm7	smhom_6xtx_1_f_5_645	Q61881	5-645
mcm7	smhom_7plo_1_g_3_645	Q61881	3-645
mcm7	smhom_6rax_1_f_3_646	Q61881	3-646
mcm7	smhom_3jc7_1_d_5_706	Q61881	5-706
mcm7	smhom_5xf8_1_d_7_716	Q61881	7-716
mcm7	smhom_3jc7_1_c_3_646	Q61881	3-646
mcm7	smhom_6hv9_1_c_5_716	Q61881	5-716
mcm7	smhom_4fdg_1_a_6_642	Q61881	6-642
met	afq8ch25-f1-model_v3	Q8CH25	1-1379
mrgprb4	afq91zc0-f1-model_v3	Q91ZC0	1-321
mrgprb4	smhom_7vv3_1_e_27_282	Q91ZC0	27-282
mtor	afq9jln9-f1-model_v3	Q9JLN9	1-2549
mtor	smhom_6zwm_1_a_17_2549	Q9JLN9	17-2549
mtor	smhom_7uxh_1_c_19_2549	Q9JLN9	19-2549
mtor	smhom_5h64_1_a_210_2549	Q9JLN9	210-2549
myof	afq69zn7-f1-model_v3	Q69ZN7	1-2048
myof	smhom_2r83_1_a_1_306	Q69ZN7	1-306
myof	smhom_4p42_1_b_1_305	Q69ZN7	1-305
nf1	smhom_7pgr_1_a_4_2747	Q04690	4-2747
nf1	smhom_7pgt_1_a_4_2747	Q04690	4-2747
nlrp12	afe9q5r7-f1-model_v3	E9Q5R7	1-1054
nlrp12	smhom_8ej4_1_a_133_970	E9Q5R7	133-970
nlrp12	smhom_8ej4_1_a_123_1053	E9Q5R7	123-1053
nlrp12	smhom_7wbt_1_a_124_1054	E9Q5R7	124-1054
nlrp12	smhom_7vtq_1_a_124_1054	E9Q5R7	124-1054
nlrp12	smhom_7pzc_1_a_10_1054	E9Q5R7	10-1054

nlrp12	smhom_7wbt_1_a_165_971	E9Q5R7	165-971
nlrp12	smhom_7pzc_1_a_12_970	E9Q5R7	12-970
nlrp12	smhom_5irm_1_a_192_845	E9Q5R7	192-845
notch2	afo35516-f1-model_v3	O35516	1-2473
notch2	smhom_3v79_1_a_1831_2069	O35516	1831-2069
notch2	smhom_2oo4_2_a_1425_1674	O35516	1425-1674
notch2	smhom_4xbm_2_a_90_297	O35516	90-297
notch2	smhom_4xbm_2_a_819_1024	O35516	819-1024
notch2	smhom_6py8_1_e_1703_2069	O35516	1703-2069
notch2	smhom_4xbm_2_a_286_531	O35516	286-531
notch2	smhom_4xbm_2_a_742_948	O35516	742-948
notch2	smhom_4xbm_2_a_732_986	O35516	732-986
notch2	smhom_4xbm_2_a_200_493	O35516	200-493
notch2	smhom_4xbm_2_a_1011_1224	O35516	1011-1224
notch2	smhom_4xbm_2_a_937_1186	O35516	937-1186
notch2	smhom_4xbm_2_a_1004_1263	O35516	1004-1263
notch3	afq61982-f1-model_v3	Q61982	1-2318
notch3	smhom_4zlp_1_a_1386_1638	Q61982	1386-1638
notch3	smhom_5uk5_1_a_278_468	Q61982	278-468
notch3	smhom_7xce_1_a_1821_2071	Q61982	1821-2071
notch3	smhom_4xbm_2_a_378_582	Q61982	378-582
notch3	smhom_6py8_1_e_1667_2027	Q61982	1667-2027
notch3	smhom_4xbm_2_a_66_274	Q61982	66-274
notch3	smhom_4xbm_2_a_302_545	Q61982	302-545
notch3	smhom_4xbm_2_a_418_620	Q61982	418-620
notch3	smhom_4xbm_2_a_986_1246	Q61982	986-1246
notch3	smhom_4xbm_2_a_43_197	Q61982	43-197
notch3	smhom_4xbm_2_a_261_469	Q61982	261-469
notch4	afp31695-f1-model_v3	P31695	1-1964
notch4	smhom_5mak_1_b_1548_1840	P31695	1548-1840
notch4	smhom_5czv_1_a_1173_1431	P31695	1173-1431
notch4	smhom_6py8_1_e_1473_1815	P31695	1473-1815
notch4	smhom_5jhq_1_a_1597_1849	P31695	1597-1849
notch4	smhom_4xbm_2_a_416_622	P31695	416-622
notch4	smhom_4xbm_2_a_611_840	P31695	611-840
notch4	smhom_4xbm_2_a_338_547	P31695	338-547
notch4	smhom_4xbm_2_a_496_725	P31695	496-725
notch4	smhom_4xbm_2_a_98_272	P31695	98-272
notch4	smhom_4xbm_2_a_216_428	P31695	216-428
nras	afp08556-f1-model_v3	P08556	1-189
nras	smhom_4dst_1_a_2_181	P08556	2-181
nup214	afq80u93-f1-model_v3	Q80U93	1-2085
nup214	smhom_3fmo_1_a_8_428	Q80U93	8-428
nup214	smhom_3vp9_1_b_803_880	Q80U93	803-880
nup214	smhom_7vop_1_0_684_897	Q80U93	684-897
opn3	afq9wuk7-f1-model_v3	Q9WUK7	1-400
opn3	smhom_5dgy_1_b_37_327	Q9WUK7	37-327
pde4dip	afq80yt7-f1-model_v3	Q80YT7	1-2224

pde4dip	smhom_7ko4_1_p_238_414	Q80YT7	238-414
pde4dip	smhom_6jlb_1_a_245_413	Q80YT7	245-413
pde4dip	smhom_7utl_1_s_484_660	Q80YT7	484-660
pde4dip	smhom_7d9n_1_c_242_416	Q80YT7	242-416
pde4dip	smhom_7utl_1_t_484_660	Q80YT7	484-660
pde4dip	smhom_7nep_1_m_238_447	Q80YT7	238-447
pde4dip	smhom_7ju4_1_a_525_686	Q80YT7	525-686
pde4dip	smhom_6x0v_1_d_6_37	Q80YT7	6-37
pde4dip	smhom_7r5k_94_a_255_399	Q80YT7	255-399
peg10	afq7tn75-f1-model_v3	Q7TN75	1-958
peg10	smhom_6s7y_1_a_133_295	Q7TN75	133-295
peg10	smhom_6r24_1_i_144_302	Q7TN75	144-302
peg10	smhom_5a9e_2_a_176_292	Q7TN75	176-292
pign	afq9r1s3-f1-model_v3	Q9R1S3	1-931
pign	smhom_5tcd_1_a_43_350	Q9R1S3	43-350
plcb1	afq9z1b3-f1-model_v3	Q9Z1B3	1-1216
plcb1	smhom_4qj3_1_b_12_833	Q9Z1B3	12-833
plcb1	smhom_3qr0_1_a_11_836	Q9Z1B3	11-836
plcb1	smhom_7t8t_1_a_17_798	Q9Z1B3	17-479
plcb1	smhom_1jad_1_b_902_1174	Q9Z1B3	902-1174
prex2	afq3lac4-f1-model_v3	Q3LAC4	1-1598
prex2	smhom_5fi0_1_a_5_359	Q3LAC4	5-359
prex2	smhom_7syf_1_a_8_1597	Q3LAC4	8-1597
prex2	smhom_7ped_1_a_466_562	Q3LAC4	466-562
prex2	smhom_4h6y_1_a_19_269	Q3LAC4	19-269
prex2	smhom_7pec_1_d_371_736	Q3LAC4	371-736
psme4	afq5ssw2-f1-model_v3	Q5SSW2	1-1843
psme4	smhom_8cvs_1_2_24_1843	Q5SSW2	24-1843
pten	afo08586-f1-model_v3	O08586	1-403
pten	smhom_7jvx_1_a_7_351	O08586	7-351
ptprt	afq99m80-f1-model_v3	Q99M80	1-1454
ptprt	smhom_2v5y_1_a_36_595	Q99M80	36-595
ptprt	smhom_6kr4_1_a_882_1454	Q99M80	882-1454
ptprt	smhom_6j6u_1_a_882_1452	Q99M80	882-1452
rasa1	afe9pyg6-f1-model_v3	E9PYG6	1-813
rasa1	smhom_8dgq_1_a_165_434	E9PYG6	20-209
rasa1	smhom_3bxj_2_a_579_1019	E9PYG6	354-794
rasa1	smhom_6pbc_1_a_166_704	E9PYG6	1-479
rb1	afp13405-f1-model_v3	P13405	1-921
rb1	smhom_4ell_2_a_378_779	P13405	378-779
rb1	smhom_4elj_1_a_47_764	P13405	47-764
rbm46	afp86049-f1-model_v3	P86049	1-533
rbm46	smhom_6kor_1_a_140_310	P86049	140-310
rbm46	smhom_7q4l_1_a_18_226	P86049	18-226
rbm46	smhom_2ghp_1_a_61_309	P86049	61-309
rbm46	smhom_6y53_1_h_59_225	P86049	59-225
rbm46	smhom_5vsu_1_a_58_310	P86049	58-310
rbm46	smhom_3vf0_1_a_58_311	P86049	58-311

ripk2	afp58801-f1-model_v3	P58801	1-539
ripk2	smhom_6fu5_1_a_5_316	P58801	5-315
ripk2	smhom_7mfe_1_a_26_314	P58801	26-314
ros1	afq78dx7-f1-model_v3	Q78DX7	1-2340
ros1	smhom_7z5w_1_a_1930_2218	Q78DX7	1930-2218
rpgrip1	afq9epq2-f1-model_v3	Q9EPQ2	1-1331
rpgrip1	smhom_4qam_1_b_1142_1328	Q9EPQ2	1142-1328
rpgrip1	smhom_2yrb_1_a_581_720	Q9EPQ2	581-720
scn10a	afq6qiy3-f1-model_v3	Q6QIY3	1-1958
scn10a	smhom_7w9k_1_a_9_1854	Q6QIY3	9-1854
scn11a	afq9r053-f1-model_v3	Q9R053	1-1765
scn11a	smhom_7w9k_1_a_12_1715	Q9R053	12-1715
scn1a	afa2apx8-f1-model_v3	A2APX8	1-2009
scn1a	smhom_6a95_1_a_1202_1901	A2APX8	1202-1901
scn1a	smhom_7w9k_1_a_10_1917	A2APX8	10-1917
scn3a	afa2asi5-f1-model_v3	A2ASI5	1-1947
scn3a	smhom_6a95_1_a_1142_1838	A2ASI5	1142-1838
scn3a	smhom_7w9k_1_a_10_1854	A2ASI5	10-1854
setd2	afe9q5f9-f1-model_v3	E9Q5F9	1-2537
setd2	smhom_2a7o_1_a_2435_2534	E9Q5F9	2435-2534
setd2	smhom_5lsx_1_a_1420_1665	E9Q5F9	1420-1665
setd2	smhom_6zv2_1_a_1689_1804	E9Q5F9	1689-1804
setx	afa2akx3-f1-model_v3	A2AKX3	1-2646
setx	smhom_5mzn_1_a_1676_2429	A2AKX3	1676-2429
sis	afp31240-f1-model_v3	P31240	1-241
sis	smhom_4hqu_1_c_88_182	P31240	88-182
slc13a1	afq9jhi4-f1-model_v3	Q9JHI4	1-594
slc13a1	smhom_7jsk_1_a_12_586	Q9JHI4	12-586
slc13a1	smhom_6wu3_1_a_419_572	Q9JHI4	419-572
slc13a1	smhom_7t9g_1_a_34_305	Q9JHI4	34-305
smarca4	afq3tkt4-f1-model_v3	Q3TKT4	1-1613
smarca4	smhom_6hax_1_a_1418_1532	Q3TKT4	1418-1532
smarca4	smhom_7y8r_1_j_365_1312	Q3TKT4	365-1312
smarca4	smhom_7vdv_1_j_167_1349	Q3TKT4	167-1349
smo	afq99k82-f1-model_v3	Q99K82	1-793
soat2	afo88908-f1-model_v3	O88908	1-525
soat2	smhom_6l47_1_a_99_523	O88908	99-523
spen	smhom_4p6q_1_a_336_618	Q62504	336-618
spen	smhom_2rt5_1_a_3476_3644	Q62504	3476-3644
spen	smhom_2ghp_1_a_336_590	Q62504	336-590
spen	smhom_6aso_1_a_332_592	Q62504	332-592
spen	smhom_3h2u_1_b_332_592	Q62504	332-592
spen	smhom_4n0t_1_a_337_592	Q62504	337-592
spen	smhom_6y53_1_h_334_515	Q62504	334-515
st18	afq80ty4-f1-model_v3	Q80TY4	1-1045
st18	smhom_7u2a_1_c_920_989	Q80TY4	920-989
st18	smhom_2mf8_1_a_764_841	Q80TY4	764-841
st18	smhom_2cs8_1_a_811_904	Q80TY4	811-904

st18	smhom_2mf8_1_a_719_793	Q80TY4	719-793
st18	smhom_2mf8_1_a_363_437	Q80TY4	363-437
sufu	afq9z0p7-f1-model_v3	Q9Z0P7	1-484
sufu	smhom_4kmd_1_a_28_480	Q9Z0P7	28-480
sufu	smhom_4kmh_1_a_1_481	Q9Z0P7	1-481
sufu	smhom_4bla_4_a_35_481	Q9Z0P7	35-481
synm	afq70iv5-f1-model_v3	Q70IV5	1-1561
synm	smhom_3uf1_1_a_54_159	Q70IV5	56-159
synm	smhom_7d9n_1_b_10_160	Q70IV5	10-160
synm	smhom_6u42_440_a_12_157	Q70IV5	12-157
synm	smhom_6jlb_1_c_7_217	Q70IV5	7-217
synm	smhom_7rro_154_a_14_161	Q70IV5	14-161
synm	smhom_6jlb_1_b_25_186	Q70IV5	25-186
taf2	afq8c176-f1-model_v3	Q8C176	1-1104
taf2	smhom_7egc_1_l_9_978	Q8C176	9-978
tas2r102	afq7m717-f1-model_v3	Q7M717	1-329
tas2r102	smhom_7xp4_1_e_5_302	Q7M717	5-302
tet2	afq4jk59-f1-model_v3	Q4JK59	1-1912
tet2	smhom_5d9y_1_a_1047_1378	Q4JK59	1047-1378
tnr	afq8byi9-f1-model_v3	Q8BYI9	1-1358
tnr	smhom_1tdq_1_a_503_771	Q8BYI9	503-771
tnr	smhom_1fnf_1_a_415_765	Q8BYI9	415-765
tnr	smhom_6mfa_1_a_503_858	Q8BYI9	503-858
tnr	smhom_3t1w_1_a_414_769	Q8BYI9	414-769
tnr	smhom_3t1w_1_a_593_950	Q8BYI9	593-950
tnr	smhom_1fnf_1_a_686_1039	Q8BYI9	686-1039
tnr	smhom_3t1w_1_a_685_1033	Q8BYI9	685-1033
tnr	smhom_1fnf_1_a_594_943	Q8BYI9	594-943
tnr	smhom_6mfa_1_a_593_947	Q8BYI9	593-947
tnr	smhom_3t1w_1_a_774_1129	Q8BYI9	774-1129
tnr	smhom_1fnf_1_a_774_1122	Q8BYI9	774-1122
tnr	smhom_3t1w_1_a_326_675	Q8BYI9	326-675
tnr	smhom_6mfa_1_a_773_1127	Q8BYI9	773-1127
tnr	smhom_6mfa_1_a_415_764	Q8BYI9	415-764
tnr	smhom_6mfa_1_a_326_680	Q8BYI9	326-680
tnr	smhom_6tpw_1_a_415_767	Q8BYI9	415-767
tnr	smhom_6tpw_1_a_594_945	Q8BYI9	594-945
tnr	smhom_2v5y_1_a_655_955	Q8BYI9	655-955
tnr	smhom_6tpw_1_a_326_678	Q8BYI9	326-678
tnr	smhom_2v5y_1_a_742_1043	Q8BYI9	742-1043
tnr	smhom_3l5h_1_a_502_944	Q8BYI9	502-944
trrap	afq80yv3-f1-model_v3	Q80YV3	1-2565
trrap	smhom_8h7g_1_a_8_2565	Q80YV3	8-2565
trrap	smhom_7kts_1_a_12_2565	Q80YV3	12-2565
trrap	smhom_7ktr_1_a_8_2565	Q80YV3	8-2565
trrap	smhom_8esc_1_a_3_2565	Q80YV3	3-2565
trrap	smhom_6t9j_1_c_3_2565	Q80YV3	3-2565
trrap	smhom_8esc_1_a_523_2044	Q80YV3	523-2044

tsc1	afq9ep53-f1-model_v3	Q9EP53	1-1161
tsc1	smhom_7dl2_1_a_743_968	Q9EP53	743-968
tsc1	smhom_7dl2_1_d_743_966	Q9EP53	743-966
tsc1	smhom_7utl_1_t_776_972	Q9EP53	776-972
tsc1	smhom_7nep_1_l_682_849	Q9EP53	682-849
tsc1	smhom_6kn7_1_p_737_972	Q9EP53	737-972
tsc1	smhom_7ko4_1_p_682_934	Q9EP53	682-934
tsc1	smhom_1qgk_1_a_6_162	Q9EP53	6-162
ttc27	afq8cd92-f1-model_v3	Q8CD92	1-847
ttc27	smhom_6eou_1_a_447_750	Q8CD92	447-750
ttc27	smhom_7dhg_1_a_439_751	Q8CD92	439-751
ttc27	smhom_7kw7_1_e_557_750	Q8CD92	557-750
ttc27	smhom_7dkh_1_a_399_750	Q8CD92	399-750
ttc27	smhom_5w5i_1_a_441_750	Q8CD92	441-750
ttc27	smhom_4g1t_1_b_435_752	Q8CD92	435-752
usp24	afb1ay13-f1-model_v3	B1AY13	1-2617
usp24	smhom_7yxx_1_a_372_2571	B1AY13	372-2571
usp24	smhom_7yxx_1_b_372_2571	B1AY13	372-2571
usp26	afq99mx1-f1-model_v3	Q99MX1	1-835
usp26	smhom_5xve_1_a_282_538	Q99MX1	282-538
usp9x	afp70398-f1-model_v3	P70398	1-2559
usp9x	smhom_7yxx_1_a_321_2475	P70398	321-2475
vmn2r81	afq80z09-f1-model_v3	Q80Z09	1-865
vmn2r81	smhom_7m3j_1_a_33_854	Q80Z09	33-853
vmn2r81	smhom_7dd5_1_a_24_847	Q80Z09	24-847
wnk1	afp83741-f1-model_v3	P83741	1-2377
wnk1	smhom_6cn9_1_a_210_480	P83741	210-480
wnk1	smhom_5o2c_1_a_212_565	P83741	212-565
zan	smhom_8d3c_1_a_1278_2398	O88799	1278-2398
zfhx3	smhom_2da3_1_a_2150_2209	Q61329	2150-2209
zfhx3	smhom_5hod_1_a_2651_2707	Q61329	2651-2707
zfhx3	smhom_2lkx_1_a_2249_2308	Q61329	2249-2308
zfhx3	smhom_1p7i_1_a_2956_3008	Q61329	2956-3008
zfp39	afq02525-f1-model_v3	Q02525	1-718
zfp39	smhom_5v3m_1_c_408_684	Q02525	408-684
zfp39	smhom_5wjq_1_c_407_683	Q02525	407-683
zfp39	smhom_5v3j_2_c_464_715	Q02525	464-715
zfp39	smhom_5v3j_2_c_436_713	Q02525	436-713
zfp39	smhom_5wjq_1_c_406_712	Q02525	406-712
zfp39	smhom_5v3j_2_c_408_713	Q02525	408-713
zfp39	smhom_5v3m_1_c_353_628	Q02525	353-628
zfp39	smhom_5v3m_1_c_295_600	Q02525	295-600
zfp39	smhom_5wjq_1_c_353_628	Q02525	353-628
zfp39	smhom_5wjq_1_c_298_600	Q02525	298-600
zfp39	smhom_5v3m_1_c_299_572	Q02525	299-572
zfp39	smhom_5v3j_2_c_295_629	Q02525	295-629
zfp750	afq8bh05-f1-model_v3	Q8BH05	1-703
zfp750	smhom_5kl5_1_a_22_46	Q8BH05	22-46

10.3 Solvent accessibly surface area

The Python package FreeSASA [79] was used to calculate the total solvent accessible surface area using the default settings.

10.4 Clinvar data

We used the the ClinVar variant_summary.txt file downloaded from the ClinVar FTP site (https://ftp.ncbi.nlm.nih.gov/pub/clinvar/) . Accessed 7th April 2020.

10.5 Monte Carlo test

Each possible single nucleotide change was enumerated for the region to be tested. For each of these potential mutations, a relative mutation rate (Methods) and a metric score, e.g. ΔΔG, was calculated. The null hypothesis of neutral selection assumed that the probability of observing each mutation, and therefore the distribution of metric scores, is determined solely by the mutational spectrum. A cumulative distribution of scores under the null hypothesis was calculated, the score for each possible mutation converted to a cumulative distribution function (CDF) score, and the sum of these CDF scores taken as the test statistic. Using the summed CDF score instead of the mean of the raw scores for the test statistic means that the test is less sensitive to extreme outlier values. Using the median would also be less sensitive to outliers than the mean, but would be inappropriate for testing some discrete metrics. The best choice of statistic to use in the test may depend on the particular data and the question being asked (for example, if the outlier mutations are crucial and reliably scored, then mean may be more appropriate). However, the CDF score sum is a robust option that works for the metric scores tested in this project**.**

Let n be the number of observed mutations in the region to test. A large number N (here N=100000) of random draws of n values from the null distribution of CDF scores was made, and for each draw, i, the sum of the CDF scores, s_i, was calculated. The sum of the CDF scores for the observed mutations, s_{obs}, was also calculated. Then, b, the number of the s_i values that were more extreme than s_{obs}, was counted. This was converted into a two-tailed p-value by multiplying the exact Monte Carlo p-value [80] by two, i.e.

$$p = 2 \times \frac{(b+1)}{N+1}$$

where

$$b = \min\left(\sum_{i=0}^{N}\begin{cases}1, & s_i \leq s_{obs} \\ 0, & s_i > s_{obs}\end{cases}, \quad \sum_{i=0}^{N}\begin{cases}0, & s_i < s_{obs} \\ 1, & s_i \geq s_{obs}\end{cases}\right)$$

The minimum p-value possible with N=100000 is just under $2e^{-5}$ (2/100001).

The central limit theorem means that, under certain conditions, the null distribution from this Monte Carlo test can be approximated by a normal distribution. However, as the normal approximation is not appropriate for small sample sizes and skewed score distributions [81], it has not been used here.

10.6 Binomial test

In the special case that there are only two possible values for the metric score (e.g. on/not on an interface), the binomial test can be used. This means very small p-values can be accurately calculated. Similar to the Monte Carlo test, the null model is calculated from the mutational spectrum, the nucleotide sequence of the gene or gene region, and the number of observed mutations. We used the Python package SciPy [82] to calculate the p-values. The method to calculate the two-tailed p-value differs slightly from the method described above for the Monte Carlo test, but otherwise the results for Monte Carlo test with infinitely large N would be equivalent to the results of the binomial test.

10.7 Confidence intervals

95% confidence intervals were calculated by taking 10000 random samples from the null distribution or by bootstrapping 10000 random samples with replacement from the observed data.

Supplementary Figures

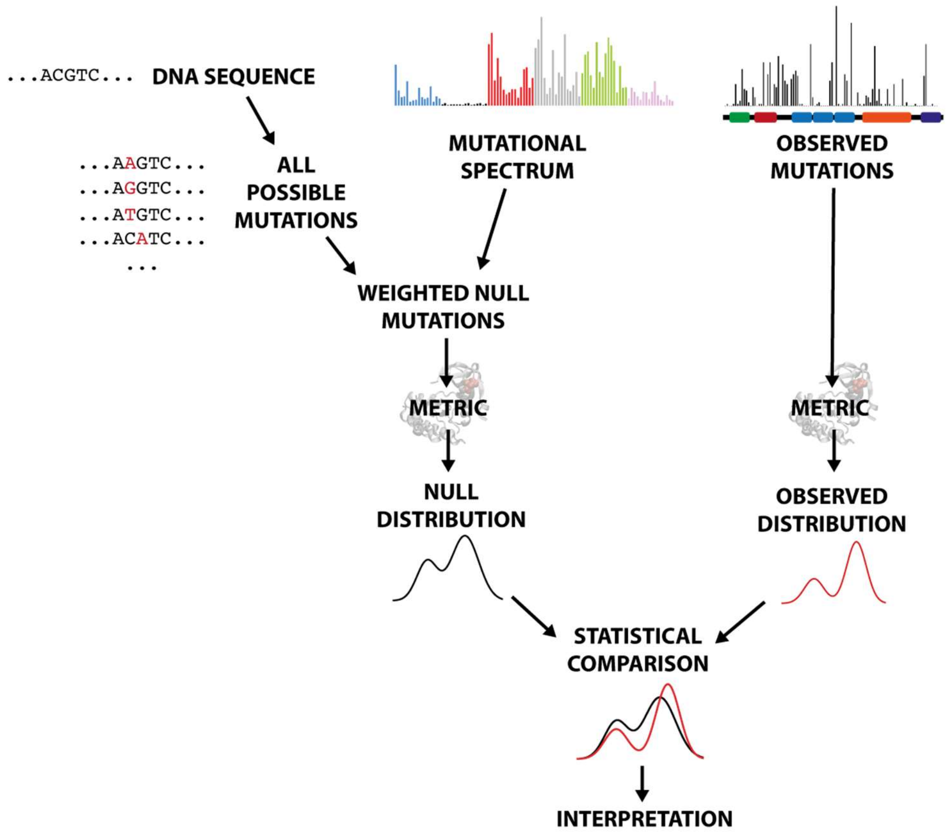

Figure S1 Schematic of the statistical test for a selected feature. The null hypothesis assumes that the mutations appear based on the mutational spectrum, and no selection or other bias is occurring. To generate the metric distribution under the null hypothesis, all possible single nucleotide mutations are generated for the gene or region to be tested, weighted by the mutational spectrum and scored using the metric. The alternative hypothesis is that some selection occurs and that it correlates with the tested metric, leading to a shift in the distribution of metric scores. The null and alternative distributions are compared using a statistical test (Supplementary Note 10). The comparison determines if the mutations with a high/low metric score are more strongly selected than the rest of the mutations in the tested region, and therefore the results of the test must be interpreted while considering other causes of selection in the region.

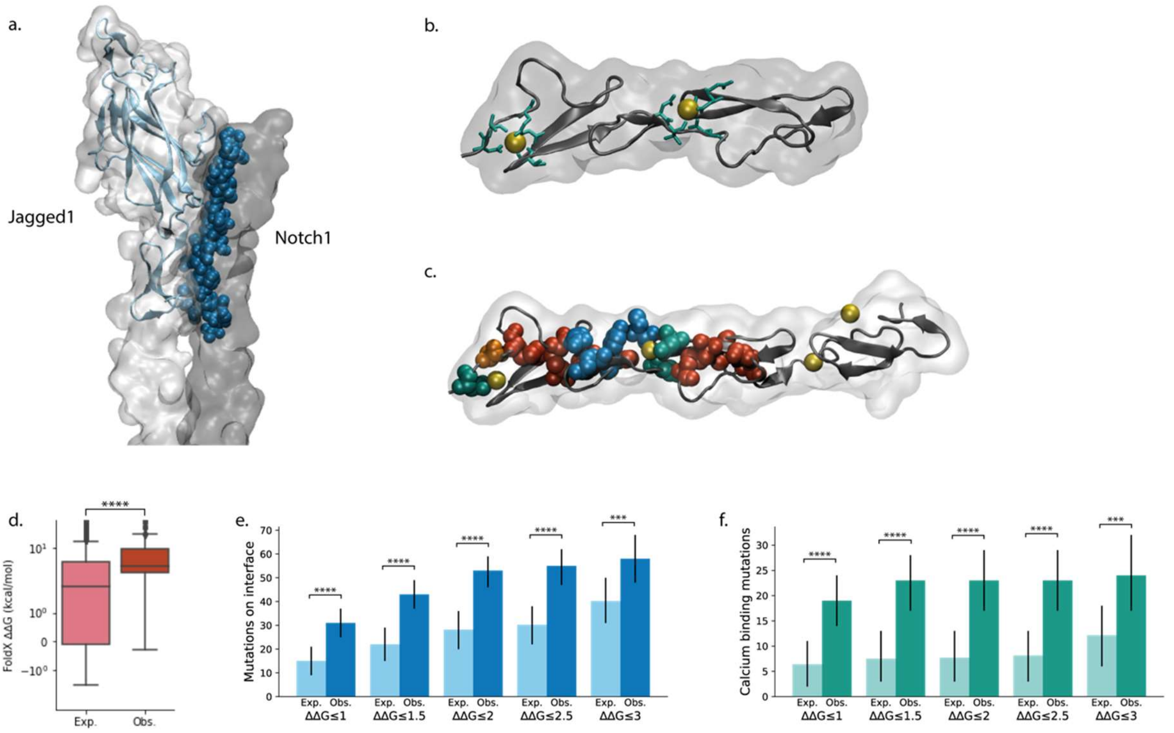

Figure S2 Selection of missense mutations in NOTCH1 EGF11–12 in normal human oesophagus. a) Ligand-binding interface residues of NOTCH1 EGF11–12 (**Error! Reference source not found.**), blue spheres, shown on rat NOTCH1 bound to JAG1 (structure 5UK5) [83]. **b)** Calcium-binding residues in NOTCH1 EGF11–12 (**Error! Reference source not found.**) shown on structure 2VJ3 [27]. Calcium-binding residues shown in green, Ca^{2+} ions shown in yellow. **c)** Residues containing at least four missense mutations in the normal oesophagus dataset shown on structure 2VJ3 [27]. Note the none of the recurrent mutations occur on EGF13 at the right-hand end of the structure. Residues coloured based on the category of the mutations on that residue: ligand-binding interface residues, blue; calcium-binding residues, green; destabilizing residues (mutations with $\Delta\Delta G$>2kcal/mol), red; D414G, orange, does not fit into the previous categories. Calcium ions are shown in yellow. **d-f)** Analysis of selection of mutation categories while excluding all mutations in the other categories. **d)** Expected (light red) and observed (dark red) distributions of $\Delta\Delta G$ excluding all mutations on the ligand-binding interface and calcium-binding residues. n=181. **e)** Expected (light blue) and observed (dark blue) number of mutations on the ligand-binding interface excluding calcium-binding residues and excluding mutations with $\Delta\Delta G$ above the thresholds shown. n=43;56;69;74;101. **f)** Expected (light green) and observed (dark green) number of mutations on calcium-binding residues excluding the ligand-binding interface and excluding mutations with $\Delta\Delta G$ above the thresholds shown. n=31;36;39;42;67. P-values calculated using a two-tailed Monte Carlo test for **d** and using a two-tailed binomial test for **e,f** (**Error! Reference source not found.**). Error bars in **e,f** show 95% confidence intervals (**Error! Reference source not found.**). ****P≤ 0.0001, ***P≤0.001.

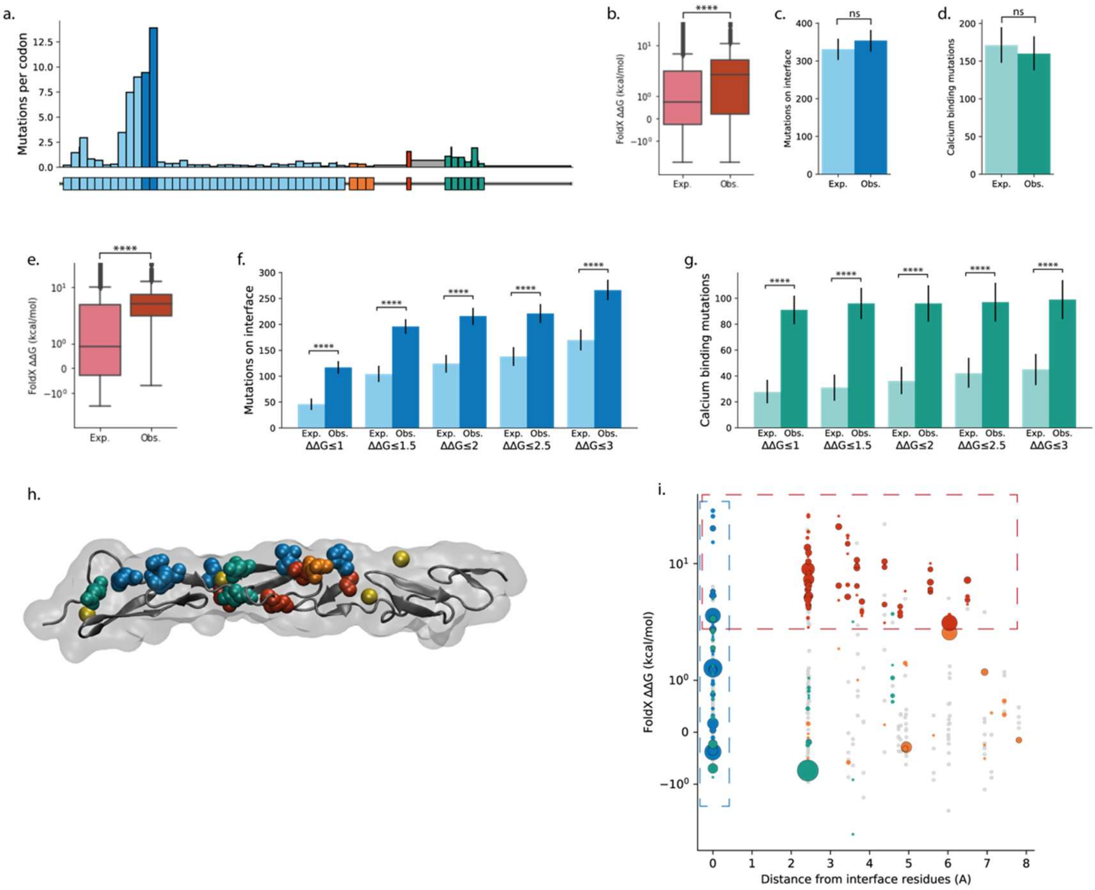

Figure S3 Selection of missense mutations in NOTCH1 EGF11–12 in normal human skin. a) Missense mutation frequency across the domains of *NOTCH1*. Domain definitions from UniProt [33]. Where the gap between domains is only a single residue, mutations from this residue are included in the subsequent domain. EGF repeats, blue; EGF11–12, dark blue; LNR repeats, orange; transmembrane region, red; ankyrin repeats, green; other regions, grey. **b)** Distributions of calculated ΔΔG values of missense mutations. Distribution expected under the neutral null hypothesis, light red, and the distribution observed, dark red. n=905. **c)** Counts of NOTCH1 EGF11–12 mutations occurring on the ligand-binding interface under the neutral null hypothesis, light blue, and observed, dark blue. Expected proportion=37%, observed=39%, n=905**. d)** Counts of NOTCH1 EGF11–12 mutations that are on calcium-binding residues under the neutral null hypothesis, light green, and observed, dark green. Expected proportion=19%, observed=18%, n=905. **e-g)** Analysis of selection of mutation categories while excluding all mutations in the other categories. **e)** Expected and observed distributions of ΔΔG excluding all mutations on the ligand-binding interface and calcium-binding residues. Colours as in **b**. n=452. **f)** Expected and observed number of mutations on the ligand-binding interface excluding calcium-binding residues and excluding mutations with ΔΔG above the thresholds shown. Colours as in **c**. n=169;260;315;360;425. **g)** Expected and observed number of mutations on calcium-binding residues excluding the ligand-binding interface and excluding mutations with ΔΔG above the thresholds shown. Colours as in **d**. n=143;160;195;236;258. **h)** Residues containing more than 10 missense mutations in skin shown on structure 2VJ3 [27]. Note the none of the recurrent mutations occur on EGF13 at the right-hand end of the structure. Residues coloured based on the category of the mutations on that residue: ligand-binding interface residues, blue; calcium-binding residues, green; destabilizing residues (mutations with ΔΔG>2kcal/mol), red; D464N, orange, does not fit into the previous categories. Calcium ions are shown in yellow. **i)** Calculated ΔΔG plotted against distance from the NOTCH1 EGF11–12 ligand-binding interface residues. Single nucleotide missense mutations that occur in the skin data set with marker size proportional to the number of times that mutation occurs shown in green if the residue is calcium binding, blue if the residue is on the ligand-binding interface, red if the mutation has ΔΔG>2kcal/mol, orange otherwise. Single nucleotide missense mutations that do not occur in the skin data set shown in grey. Regions containing highly destabilising mutations (ΔΔG>2kcal/mol) and mutations on the ligand-binding interface shown with dashed red and blue boxes respectively. P-values calculated using a two-tailed Monte Carlo test for **b**,**e** and using a two-tailed binomial test for **c,d,f,g** (**Error! Reference source not found.**). Error bars in **c,d,f,g** show 95% confidence intervals (**Error! Reference source not found.**). ****P≤ 0.0001, ns P>0.05. This data has previously been analysed using this method in the original publication of the dataset [16].

Table S1 Statistical tests of selection in *NOTCH1* EGF11–12. Tests either include all missense mutations in the region or exclude mutations in other selected categories from both the null model and observed data. FoldX ΔΔG p-values are calculated using a two-tailed Monte Carlo test; ligand-binding and calcium-binding p-values are calculated using a two-tailed binomial test (**Error! Reference source not found.**).

Tested feature	Tissue	Excluded mutations	n	Expected proportion	Observed proportion	P-value
FoldX ΔΔG	Skin	None	905	-	-	$2e^{-5}$
		Ligand-binding and calcium-binding	452	-	-	$2e^{-5}$
	Oesophagus	None	308	-	-	$2e^{-5}$
		Ligand-binding and calcium-binding	181	-	-	$2e^{-5}$
Ligand-binding interface	Skin	None	905	37%	39%	0.12
		ΔΔG>2kcal/mol and calcium-binding	315	39%	69%	$2.4e^{-25}$
	Oesophagus	None	308	35%	33%	0.72
		ΔΔG>2kcal/mol and calcium-binding	69	41%	77%	$1.7e^{-9}$
Calcium-binding	Skin	None	905	19%	18%	0.37
		ΔΔG>2kcal/mol and ligand-binding	195	18%	49%	$2.2e^{-22}$
	Oesophagus	None	308	16%	14%	0.48
		ΔΔG>2kcal/mol and ligand-binding	39	20%	59%	$8.4e^{-8}$

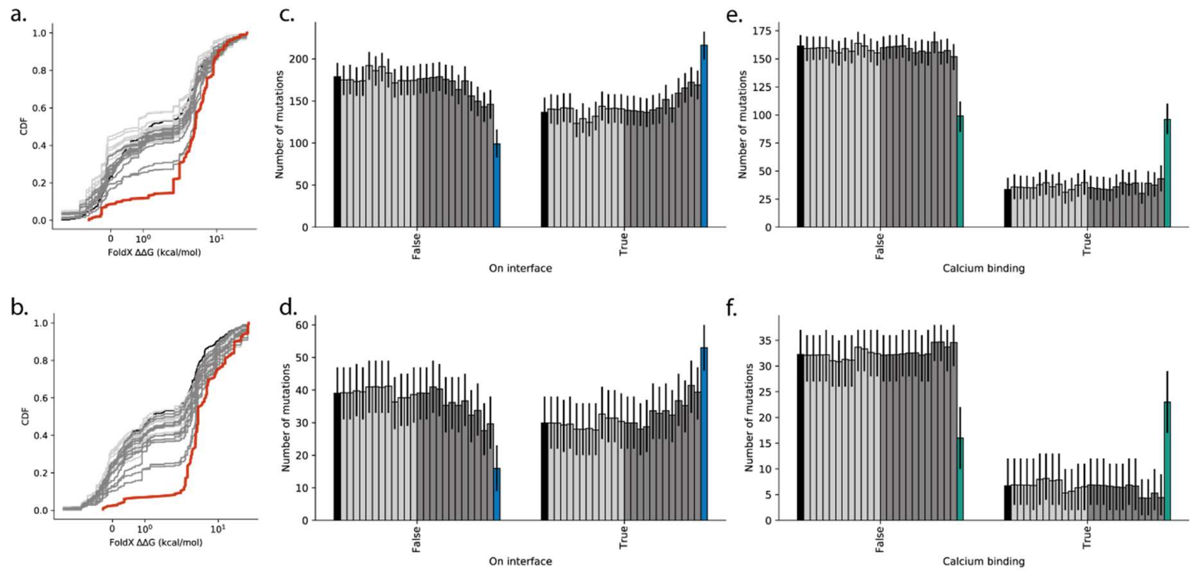

Figure S4 Effect of mutational spectrum assumptions on statistical tests of selection in NOTCH1 EGF11–12. a,b) Cumulative distributions of the observed ΔΔG values, red, and the null distributions, grey and black, for missense mutations in NOTCH1 EGF11–12. Mutations on the ligand-binding interface and calcium-binding residues excluded from both the null and observed distributions. The observed distributions are significantly different from all shown null distributions: **a)** skin, $p<0.0001$, n=452; **b)** oesophagus, $p<0.004$, n=181; two-tailed Monte Carlo test, **MethodsError! Reference source not found.**. Null distribution using a spectrum with all mutations with equal probability shown in black, null distributions with spectra calculated from mutations in all genes in the dataset shown in light grey, and null distributions with spectra calculated only from mutations in NOTCH1 shown in dark grey. **c,d)** Counts of NOTCH1 EGF11–12 missense mutations on the ligand-binding interface under the null hypotheses, grey or black, and observed, blue. Mutations with ΔΔG>2kcal/mol and mutations on calcium-binding residues are excluded. Colours of null hypotheses as in **a**. Error bars show 95% confidence intervals (**Methods**). The observed counts are significantly different from all shown null models: **c)** skin, $p<5e^{-7}$, n=315; **d)** oesophagus, $p<0.005$, n=69; two-tailed binomial test, **Methods. e,f)**. Counts of NOTCH1 EGF11–12 missense mutations on calcium-binding residues under the null hypotheses, grey or black, and observed, green. Mutations with ΔΔG>2kcal/mol and mutations on the ligand-binding interface are excluded. Colours of null hypotheses as in **a**. The observed counts are significantly different from all shown null models: **e)** skin, $p<2e^{-16}$, n=195; **f)** oesophagus, $p<3e^{-7}$, n=39; two-tailed binomial test, **Methods**. Error bars show 95% confidence intervals (**Methods**). Full list of p-values shown in

Table **S2**.

Table S2 P-values of statistical tests of selection in NOTCH1 EGF11–12 under null models that use different assumptions for the mutational spectrum. For each test, mutations in the other two categories (ΔΔG>2kcal/mol, ligand-binding, or calcium-binding) were excluded from both the null model and the observed data. The "Even" spectrum assigns an equal probability to every mutation. The "global" spectra calculate the mutation rates using all exonic single nucleotide mutations in the dataset. The "transcript" spectra calculate the mutation rates using only the exonic single nucleotide mutations in *NOTCH1*. The number in the spectrum name is the number of mutation rates in the spectrum. "6" and "12" do not use a wider nucleotide context, "96" and "192" use a trinucleotide context, and "1536" and "3072" use pentanucleotide contexts. "12", "192" and "3072" distinguish between the transcribed and non-transcribed strands, while "6", "96" and "1536" do not. "dedup" indicates that duplicate mutations (defined as having the same chromosomal position, reference base and mutant base) are removed before calculating the spectrum. The two-tailed Monte Carlo test was used for the ΔΔG tests, the two-tailed binomial test was used for the ligand-binding and calcium-binding tests (**Methods**). The lowest possible p-value from the Monte Carlo tests used was $2e^{-5}$. The global_192 spectrum (shaded) has been used throughout chapters 3 and 4 unless otherwise specified.

	Skin			Oesophagus		
**Spectrum**	**ΔΔG**	**Ligand-binding**	**Calcium-binding**	**ΔΔG**	**Ligand-binding**	**Calcium-binding**
**Even**	$2e^{-5}$	$2e^{-19}$	$2e^{-24}$	$2e^{-5}$	$3e^{-8}$	$6e^{-9}$
**global_6**	$2e^{-5}$	$1e^{-17}$	$2e^{-22}$	$2e^{-5}$	$3e^{-8}$	$9e^{-9}$
**global_6_dedup**	$2e^{-5}$	$7e^{-18}$	$1e^{-22}$	$2e^{-5}$	$3e^{-8}$	$9e^{-9}$
**global_12**	$2e^{-5}$	$6e^{-17}$	$4e^{-23}$	$2e^{-5}$	$1e^{-8}$	$8e^{-9}$
**global_12_dedup**	$2e^{-5}$	$2e^{-17}$	$3e^{-23}$	$2e^{-5}$	$1e^{-8}$	$7e^{-9}$
**global_96**	$2e^{-5}$	$6e^{-26}$	$9e^{-21}$	$2e^{-5}$	$2e^{-9}$	$2e^{-7}$
**global_96_dedup**	$2e^{-5}$	$5e^{-23}$	$3e^{-19}$	$2e^{-5}$	$1e^{-9}$	$2e^{-7}$
**global_192**	$2e^{-5}$	$2e^{-25}$	$2e^{-22}$	$2e^{-5}$	$2e^{-9}$	$8e^{-8}$
**global_192_dedup**	$2e^{-5}$	$1e^{-21}$	$2e^{-20}$	$2e^{-5}$	$1e^{-9}$	$1e^{-7}$
**global_1536**	$2e^{-5}$	$3e^{-16}$	$4e^{-27}$	$2e^{-5}$	$7e^{-7}$	$5e^{-11}$
**global_1536_dedup**	$2e^{-5}$	$2e^{-17}$	$1e^{-24}$	$2e^{-5}$	$2e^{-7}$	$2e^{-10}$
**global_3072**	$2e^{-5}$	$2e^{-17}$	$7e^{-21}$	$2e^{-5}$	$2e^{-7}$	$2e^{-9}$
**global_3072_dedup**	$2e^{-5}$	$1e^{-17}$	$3e^{-19}$	$2e^{-5}$	$4e^{-8}$	$4e^{-9}$
**transcript_6**	$2e^{-5}$	$2e^{-18}$	$3e^{-23}$	$2e^{-5}$	$3e^{-8}$	$9e^{-9}$
**transcript_6_dedup**	$2e^{-5}$	$1e^{-18}$	$9e^{-24}$	$2e^{-5}$	$3e^{-8}$	$8e^{-9}$
**transcript_12**	$2e^{-5}$	$7e^{-19}$	$3e^{-24}$	$2e^{-5}$	$2e^{-9}$	$7e^{-9}$
**transcript_12_dedup**	$2e^{-5}$	$2e^{-19}$	$9e^{-25}$	$2e^{-5}$	$4e^{-9}$	$4e^{-9}$
**transcript_96**	$2e^{-5}$	$4e^{-18}$	$2e^{-22}$	$2e^{-5}$	$3e^{-6}$	$3e^{-9}$
**transcript_96_dedup**	$2e^{-5}$	$4e^{-17}$	$3e^{-19}$	$2e^{-5}$	$1e^{-6}$	$2e^{-9}$
**transcript_192**	$2e^{-5}$	$3e^{-13}$	$1e^{-20}$	$2e^{-5}$	$3e^{-6}$	$9e^{-9}$
**transcript_192_dedup**	$2e^{-5}$	$3e^{-17}$	$2e^{-19}$	$2e^{-5}$	$7e^{-7}$	$5e^{-9}$
**transcript_1536**	$2e^{-5}$	$1e^{-10}$	$7e^{-28}$	$2e^{-5}$	$8e^{-5}$	$8e^{-13}$
**transcript_1536_dedup**	$2e^{-5}$	$9e^{-9}$	$1e^{-19}$	$2e^{-5}$	$2e^{-5}$	$6e^{-13}$
**transcript_3072**	$8e^{-5}$	$5e^{-7}$	$7e^{-21}$	0.004	0.004	$5e^{-11}$
**transcript_3072_dedup**	$4e^{-5}$	$9e^{-8}$	$1e^{-16}$	$4e^{-5}$	0.0009	$1e^{-12}$

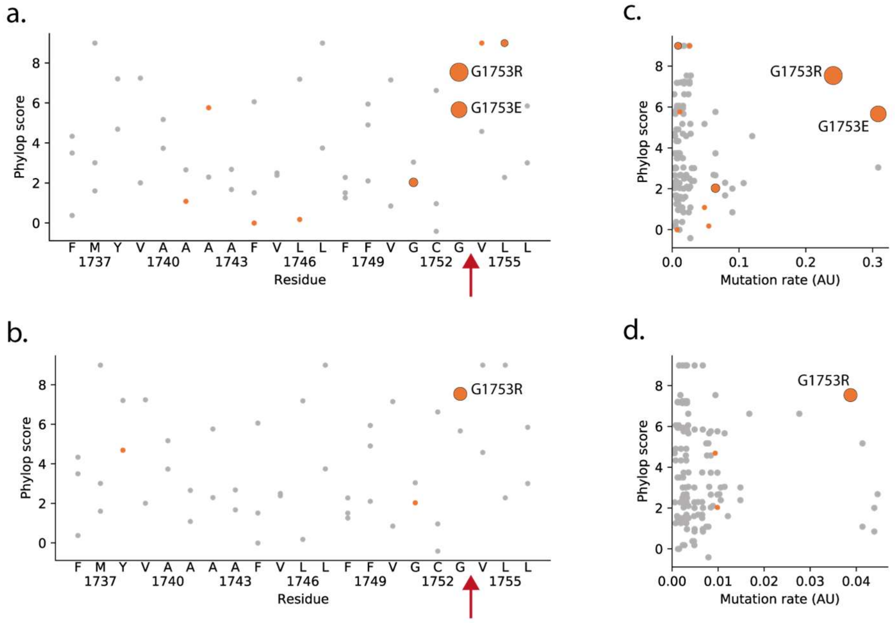

Figure S5 Missense mutations in the transmembrane helix of NOTCH1. a,b) Conservation scores from Phylop2 shown on y-axis (**Error! Reference source not found.**). Single nucleotide missense mutations that occur in the normal human skin (**a**) and oesophagus (**b**), orange, with marker size proportional to the number of times that mutation occurs. Single nucleotide missense mutations that do not occur in the dataset shown in grey. Location of the key S3-V cleavage site indicated by a red arrow. **c,d)** Phylop2 conservation score plotted against relative expected mutation rate (AU=arbitrary units) for missense mutations in the transmembrane helix of NOTCH1 for skin (**c**) and oesophagus (**d**). Single nucleotide missense mutations that occur in the dataset, orange, with marker size proportional to the number of times that mutation occurs. Single nucleotide missense mutations that do not occur shown in grey. The hotspot mutations (G1753R/E) are both highly conserved and have a high expected mutation rate.

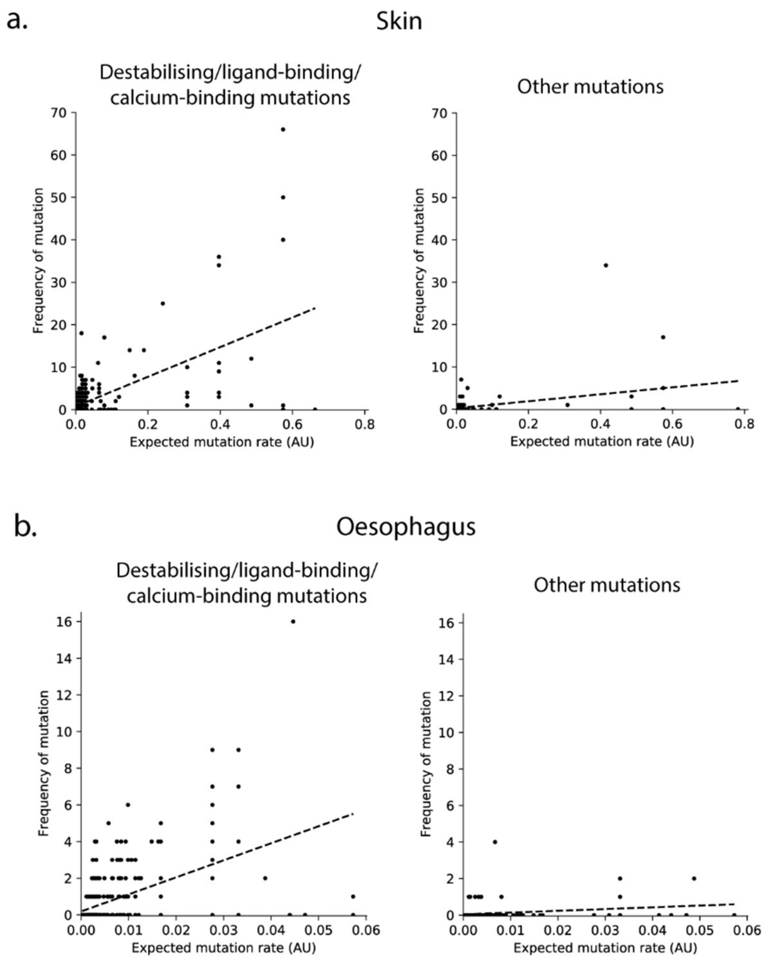

Figure S6 Expected and observed mutation rates. Expected mutation rate (**Error! Reference source not found.**, AU=arbitrary units) vs number of observations for each potential mutation in *NOTCH1* EGF11–12 in normal human skin (**a**) and oesophagus (**b**). Destabilising mutations (ΔΔG>2kcal/mol), ligand-binding mutations and calcium-binding mutations shown together on the left-hand scatter plots, all other mutations shown on the right. There is a trend of increasing number of occurrences with higher expected mutation rate (skin, destabilising, ligand-binding and calcium-binding: R^2=0.36, p-value=$3e^{-36}$; skin, other: R^2=0.13, p-value=$5e^{-6}$; oesophagus, destabilising, ligand-binding and calcium-binding: R^2=0.24, p-value=$4e^{-23}$; oesophagus, other: R^2=0.05, p-value=0.007; two-tailed Wald Test for zero slope using SciPy stats.linregress [82]). However, the observed frequency of individual mutations is not very predictable based on only the expected mutation rate and the functional impact category.

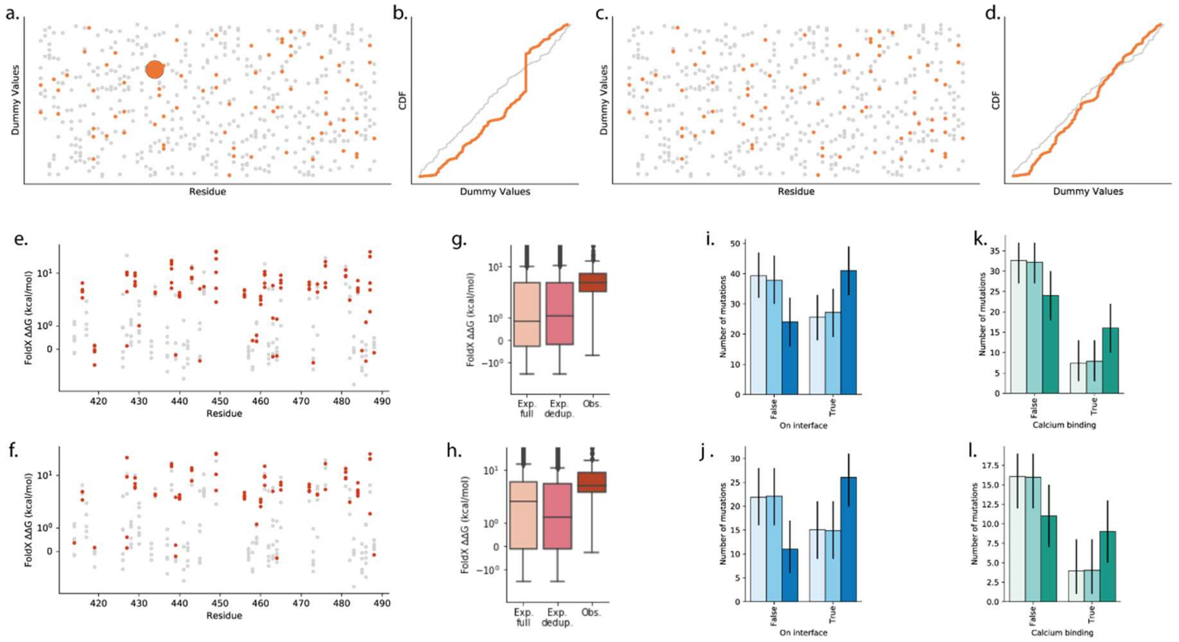

Figure S7 The effect of hotspot mutations. a-d) Demonstration of how a hotspot mutation can lead to rejection of the null hypothesis even when the metric has no biological meaning. A synthetic set of mutations was generated, where 74 mutations appear once, and one hotspot mutation appears 25 times. In place of a metric based on a biological feature, random scores between zero and one are generated for each mutation. **a)** Scatter plot of the example mutations. "Observed" mutations shown in orange, "unobserved" mutations in the region shown in grey. The hotspot mutation is the large orange marker. **b)** Cumulative distributions of the metric for the null model, grey, and the "observed" mutations. The large vertical jump in the "observed" CDF is caused by the hotspot mutation. The "observed" distribution is significantly different from the null (p=0.005, two-tailed Monte Carlo test, **Methods**). **c)** Scatter plot of the example mutations after removing duplicate mutations. "Observed" mutations shown in orange, "unobserved" mutations in the region shown in grey. The hotspot mutation is now only counted once. **d)** Cumulative distributions of the metric for the null model, grey, and the "observed" mutations after removing duplicate mutations (p=0.3, two-tailed Monte Carlo test, **Methods**). **e-l)** Reanalysis of the selection of features in NOTCH1 EGF11–12 after removing duplicate mutations. **g-l**, two null models are tested, either using the full set of mutations to calculate the mutation spectrum (lightest shade, leftmost bar/boxplot) or deduplicating the mutations prior to calculating the spectrum (middle shade, middle bar/boxplot). Observed mutations shown in the darkest shade, rightmost bar/boxplot. **e-h)** ΔΔG of mutations in NOTCH1 EGF11–12. All mutations on the ligand-binding interface or calcium-binding residues excluded from both the null model and observed data. Single nucleotide missense mutations that occur in the skin (**e**) and oesophagus (**f**) datasets, red. Single nucleotide missense mutations that do not occur in the data shown in grey. **g,h)** Distribution of calculated ΔΔG values of missense mutations in skin (**g**) and oesophagus (**h**). Distribution expected under the neutral null hypothesis using all mutations for the spectrum, left, distribution expected under the neutral null hypothesis using deduplicated mutations for the spectrum, middle, and the distribution observed after removing duplicate mutations, right. Skin: p<2e-5 for both null models, n=113; oesophagus: p<2e-5 for both null models, n=76; two-tailed Monte Carlo test (**Methods**). **i,j)** Expected and observed counts of mutations on or off the ligand-binding interface of NOTCH1 EGF11–12, where mutations with a calculated ΔΔG>2 kcal/mol and mutations on calcium-binding residues have been excluded. Skin: p<0.0007 for both null models, n=65; oesophagus: p<0.0004 for both null models, n=37; two-tailed binomial test (**Methods**). Error bars show 95% confidence intervals (**Methods**). **k,l)** Expected and observed counts of mutations on calcium-binding residues in NOTCH1 EGF11–12 where mutations with a calculated ΔΔG>2 kcal/mol or on the ligand-binding interface have been excluded. Skin: p<0.005 for both null models, n=40; oesophagus: p<0.01 for both null models, n=20; two-tailed binomial test (**Methods**). Error bars show 95% confidence intervals (**Methods**).

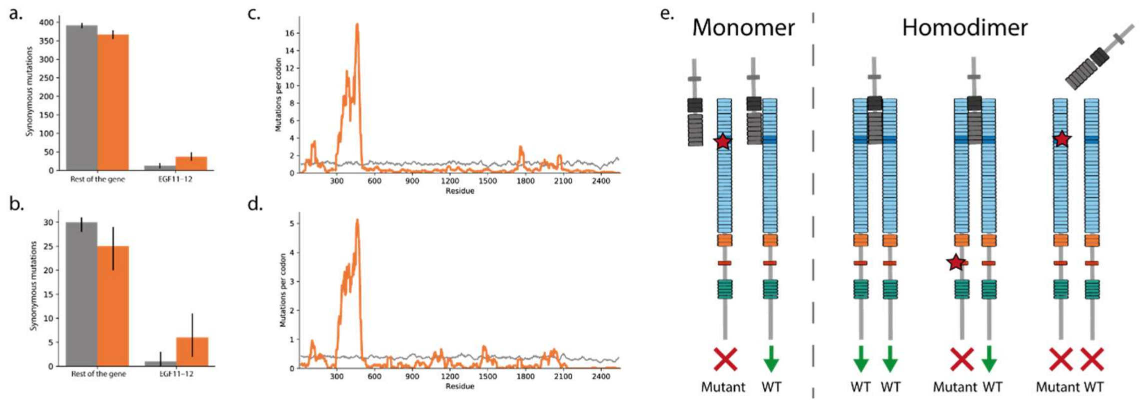

Figure S8 Potential causes of uneven missense mutation distribution across *NOTCH1*. a,b) The counts of synonymous mutations expected under the neutral null model, grey, and observed, orange, in EGF11–12 in skin (**a**) and oesophagus (**b**) (skin: $p=5.6e^{-9}$, n=404; oesophagus: p=0.0005, n=31; two-tailed binomial test, **Error! Reference source not found.**). Error bars show 95% confidence intervals (**Error! Reference source not found.**). **c,d)** Sliding windows showing the distribution of missense mutations across *NOTCH1* expected under the neutral null model, grey, and observed, orange, in skin (**c**) and oesophagus (**d**). **e)** Potential impact of mutations if NOTCH1 (shown with domains as in **Error! Reference source not found.**a) binds to the ligand (grey) as a monomer or a homodimer. If NOTCH1 binds to the ligand as a monomer (left), then a mutation in the ligand-binding EGF repeats may entirely stop signalling from the mutant allele (red cross) but the wild type (WT) allele still functions normally (green arrow). If NOTCH1 binds the ligand as a homodimer (right), then a mutant away from the ligand-binding region may stop signalling from the mutant allele but not affect the WT allele. However, a mutant in the ligand-binding region may prevent the ligand from binding, and therefore stop signalling from the WT allele as well. If NOTCH1 binds to the ligand in pairs, a heterozygous *NOTCH1* mutant would mean only one quarter of NOTCH1 pairs would be WT-WT, so a mutant that prevents ligand binding could reduce *NOTCH1* signalling by three-quarters.

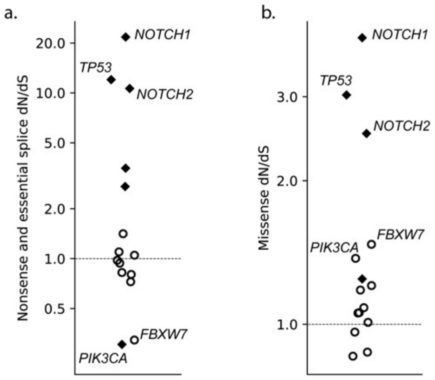

Figure S9: dN/dS ratios of genes sequenced in skin. Only genes for which high resolution protein structures containing at least 50 missense mutations in the skin data set shown (methods). The dashed line at dN/dS=1 represents neutral selection. Genes under significant selection (multiple-test corrected p-value < 0.05) shown as filled diamonds; other genes shown with open circles. The five genes analysed in detail in this study (labelled) either have very high or low nonsense dN/dS ratios, indicating selection for or against loss-of-function mutations respectively.

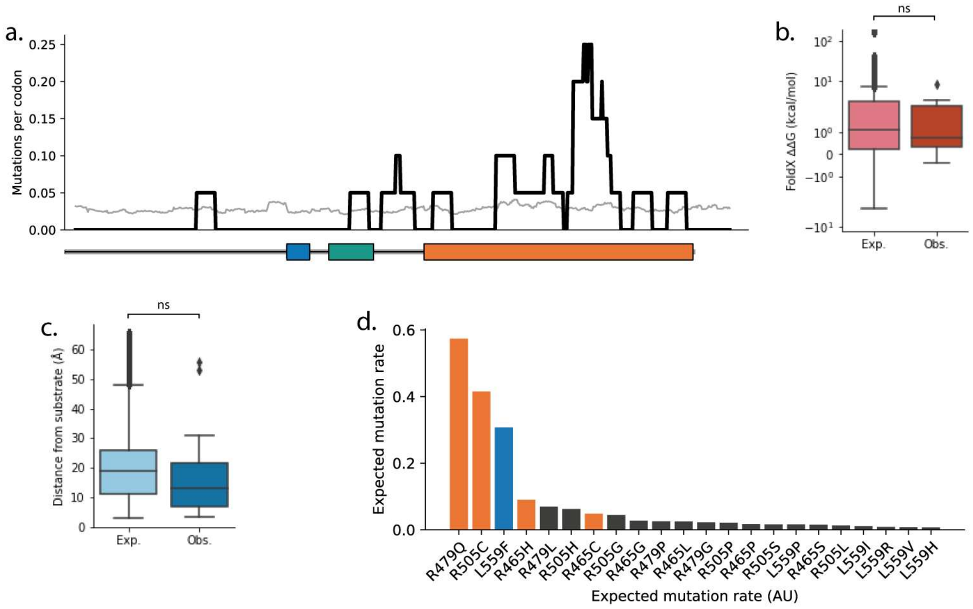

Figure S10 Missense mutations in *FBXW7*. a) Sliding window of missense mutation frequency across *FBXW7* in oesophagus. Observed distribution shown with a bold black line, the expected distribution based on the mutational spectrum shown with a thin grey line. Dimerization domain, blue bar; F-box domain, green bar; WD40 domain, orange bar. **b)** Distribution of calculated ΔΔG values of missense mutations in oesophagus. Distribution expected under the neutral null hypothesis, light red, and the distribution observed, dark red. **c)** Distribution of distances of missense mutations in oesophagus from the FBXW7 substrate (in this instance a 12-residue section of Cyclin E in PDB 2OVQ [30]). Distribution expected under the neutral null hypothesis, light blue, and the distribution observed, dark blue. **d)** Expected relative mutation rates (arbitrary units) in skin of single nucleotide missense mutations on the residues R465, R479, R505 and L559. The four most common mutations in T-ALL and CLL are shown in orange, and the most common mutation in normal skin shown in blue. P-values in **b,c** calculated using the Monte Carlo test (**Error! Reference source not found.**). ns P>0.05.

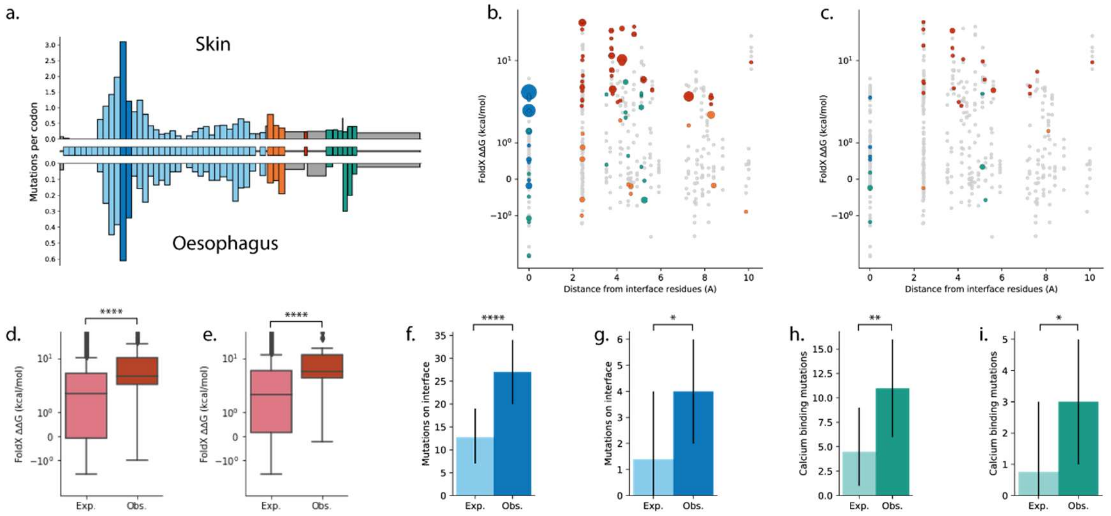

Figure S11 Selected features of missense mutations in NOTCH2 EGF11–12. **a)** Missense mutation frequency across the domains of *NOTCH2* in normal skin (top) and normal oesophagus (bottom). Domain definitions from UniProt [33]. Where the gap between domains is only a single residue, mutations from this residue are included in the subsequent domain. EGF repeats, blue; EGF11–12, dark blue; LNR repeats, orange; transmembrane region, red; ankyrin repeats, green; other regions, grey. **b,c)** Calculated ΔΔG plotted against distance from the NOTCH2 EGF11–12 ligand-binding interface for skin (**b**) and oesophagus (**c**). Single nucleotide missense mutations that occur in the dataset, with marker size proportional to the number of times that mutation occurs, shown in green if the residue is calcium binding, blue if the residue is on the ligand-binding interface, red if the mutation has ΔΔG>2kcal/mol, orange otherwise. Single nucleotide missense mutations that do not occur in the dataset shown in grey. **d,e)** Distribution of calculated ΔΔG values of missense mutations after excluding mutations on the ligand-binding interface and calcium-binding residues. Distribution expected under the neutral null hypothesis, light red, and the distribution observed, dark red. **f,g)** Counts of NOTCH2 EGF11–12 mutations occurring on the ligand-binding interface, having excluded destabilising mutations (with calculated ΔΔG > 2 kcal/mol) and calcium-binding mutations, in skin (**f**) and oesophagus (**g**). Expected counts under the neutral null hypothesis, light blue; counts observed, dark blue. **h,i)** Counts of NOTCH2 EGF11–12 mutations occurring on the calcium-binding residues, having excluded destabilising mutations (with calculated ΔΔG > 2 kcal/mol) and ligand-binding mutations, in skin (**h**) and oesophagus (**i**). Expected counts under the neutral null hypothesis, light green; counts observed, dark green. P-values calculated using a two-tailed Monte Carlo test for **d,e** and using a two-tailed binomial test for **f-i** (**Error! Reference source not found.**). Error bars in **f-i** show 95% confidence intervals (**Error! Reference source not found.**). ****P≤0.0001, **P≤0.01, *P≤0.05.

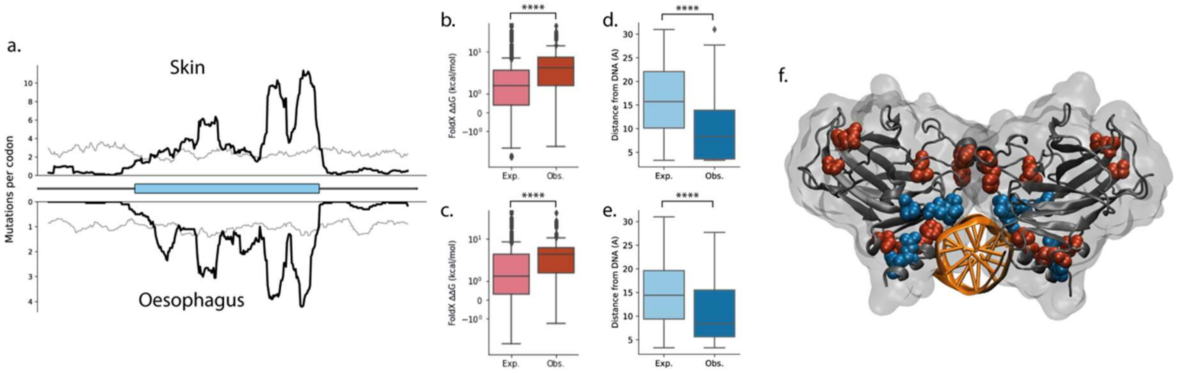

Figure S12 Selection of missense mutations in *TP53*. a) Sliding window of missense mutation frequency across *TP53* in skin (top) and oesophagus (bottom). Observed distribution shown with a bold black line, the expected distribution based on the mutational spectrum shown with a thin grey line. DNA-binding domain shown as a blue bar. Domain definition from UniProt [33]. **b,c)** Distribution of calculated ΔΔG values of missense mutations in skin (**b**) and oesophagus (**c**) after excluding mutations within 5Å of the DNA molecule. Distribution expected under the neutral null hypothesis, light red, and the distribution observed, dark red. **d,e)** Distribution of distances of missense mutations from DNA in skin (**d**) and oesophagus (**e**) after excluding mutations with ΔΔG > 2 kcal/mol. Distribution expected under the neutral null hypothesis, light blue, and the distribution observed, dark blue. **f)** Structure of the p53 DNA-binding domain bound to DNA (orange). Residues containing missense mutations that occur at least 10 times in the skin data set are highlighted. Highly destabilizing mutations (ΔΔG > 2 kcal/mol) shown in red. Non-destabilizing mutations are shown in blue. ****P≤0.0001, two-tailed Monte Carlo test, **Error! Reference source not found.**. The skin *TP53* data has previously been analysed using this method in the original publication of the dataset [16].

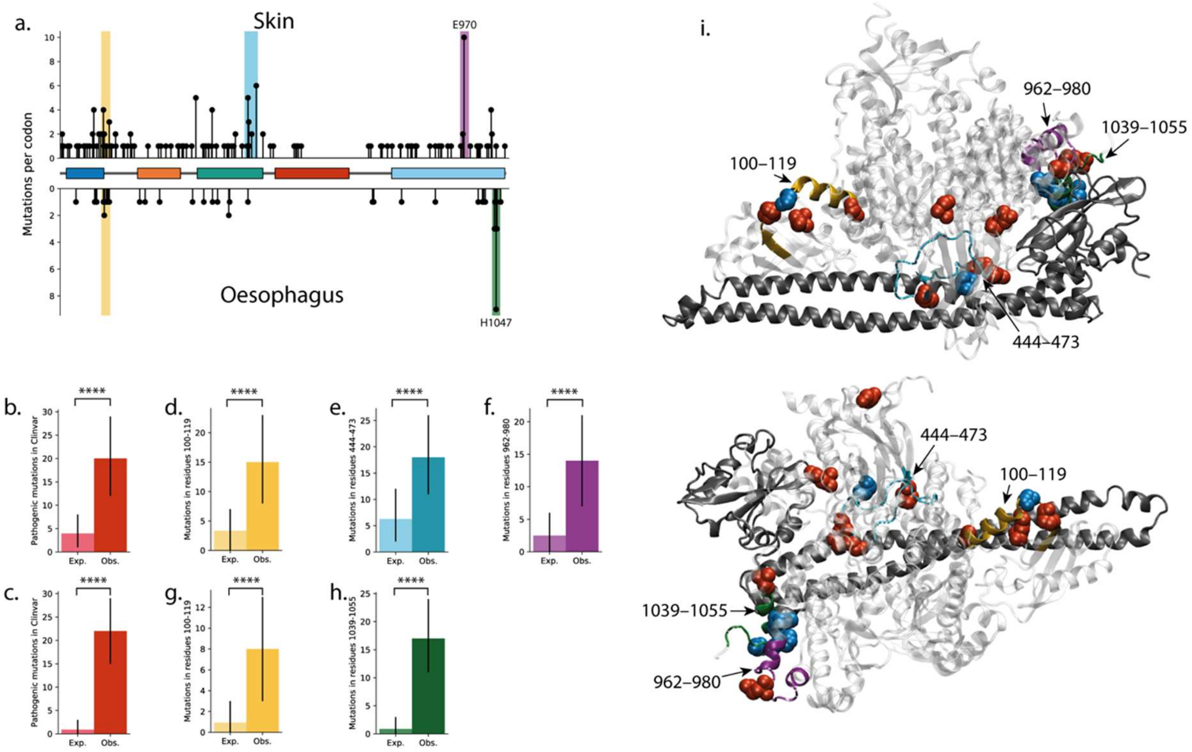

Figure S13 Selection of missense mutations in *PIK3CA*. a) Missense mutations in normal skin (top) and oesophagus (bottom). The most commonly mutated residues are labelled. Domain definitions from UniProt [33]. ABD-domain, dark blue; RBD-domain, orange; C2 domain, green; helical domain, red; kinase domain, light blue. Shaded areas show the regions with significant enrichment of missense mutations as shown in **d-i**. Residues 100–119, yellow; residues 444–473, blue; residues 962–980, purple; residues 1039–1055, green. **b,c)** Missense mutations annotated as Likely Pathogenic/Pathogenic in Clinvar [24] in skin (**b**) and oesophagus (**c**). **d-h)** Missense mutations in regions of *PIK3CA* in skin (**d-f**) and oesophagus (**g-h**). Expected counts under the neutral null hypothesis, left bar; counts observed, right bar. **i)** Structure of PI3K. p110α, white; p85α, grey. p110α residues 100–119 shown in yellow, 444–473 shown in cyan, 962–980 shown in purple and 1039–1055 shown in green. Residues mutated at least three times in the skin dataset shown as red spheres. Residues mutated at least twice in the oesophagus dataset shown as blue spheres. Error bars in **b-h** show 95% confidence intervals (**Error! Reference source not found.**). ****$P \leq 0.0001$, two-tailed binomial test, **Error! Reference source not found.**.

Table S3 Tests for enrichment of missense mutations in *PIK3CA* regions in normal skin and oesophagus. Regions based on [25]. N=total missense mutations in test, Expected=percentage of missense mutations expected on the given residues under the neutral null hypothesis, Observed=percentage of missense mutations observed on the given residues, P=two-tailed binomial p-value, **Error! Reference source not found.**, Q=Benjamini–Hochberg multiple-test corrected p-value [84]. Shaded rows show statistically significant results.

**Skin**					
**Residues**	**N**	**Expected**	**Observed**	**P**	**Q**
**100–119**	167	2%	9%	$2e^{-6}$	$9e^{-6}$
**120–127**	167	1%	1%	0.3	0.5
**335–342**	167	1%	0%	0.6	0.7
**343–350**	167	1%	2%	0.1	0.3
**444–473**	167	4%	11%	$6e^{-5}$	0.0002
**532–551**	167	2%	0%	0.06	0.2
**720–744**	167	2%	1%	0.2	0.4
**848–859**	167	1%	1%	0.7	0.7
**930–956**	167	2%	1%	0.5	0.6
**962–980**	167	1%	8%	$3e^{-7}$	$3e^{-6}$
**1039–1055**	167	1%	1%	0.7	0.7
**Oesophagus**					
**Residues**	**N**	**Expected**	**Observed**	**P**	**Q**
**100–119**	49	2%	16%	$4e^{-6}$	$2e^{-5}$
**120–127**	49	1%	0%	1	1
**335–342**	49	1%	0%	1	1
**343–350**	49	1%	2%	0.4	1
**444–473**	49	3%	2%	1	1
**532–551**	49	2%	0%	1	1
**720–744**	49	2%	0%	1	1
**848–859**	49	2%	0%	1	1
**930–956**	49	3%	0%	0.6	1
**962–980**	49	1%	0%	1	1
**1039–1055**	49	2%	35%	$7e^{-18}$	$8e^{-17}$

Table S4 Tests for enrichment of missense mutations in *PIK3CA* regions in normal skin and oesophagus, after deduplicating recurrent mutations and while excluding other selected regions in the tissue. Only significant regions from

Table **S3** tested. N=total missense mutations in test, Expected=percentage of missense mutations expected on the given residues under the neutral null hypothesis, Observed=percentage of missense mutations observed on the given residues, P=two-tailed binomial p-value, **Error! Reference source not found.**. Shaded rows show statistically significant results.

**Skin**				
**Residues**	**N**	**Expected**	**Observed**	**P**
**100–119**	114	2%	9%	0.0002
**444–473**	112	4%	7%	0.08
**962–980**	109	2%	5%	0.03
**Oesophagus**				
**Residues**	**N**	**Expected**	**Observed**	**P**
**100–119**	30	2%	23%	$1e^{-6}$
**1039–1055**	31	2%	26%	$6e^{-8}$

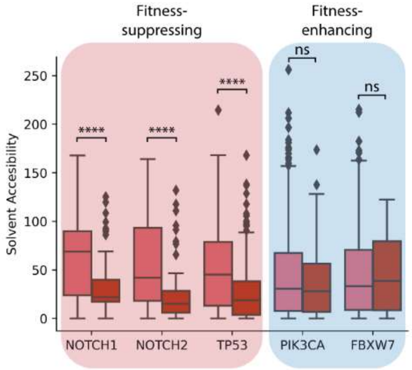

Figure S14 Solvent-accessibility of missense mutations. Expected (light red) and observed (dark red) distributions of solvent accessible surface area of mutated residues (methods). Residues on critical functional sites have been excluded from both the expected and observed distributions. Functional sites are defined as the ligand-binding interface and calcium-binding residues for NOTCH1 and NOTCH2, residues within 8Å of DNA or substrate for TP53 and FBXW7 respectively, and residues in the regions 100–119, 444–473, 962–980 for *PIK3CA*.

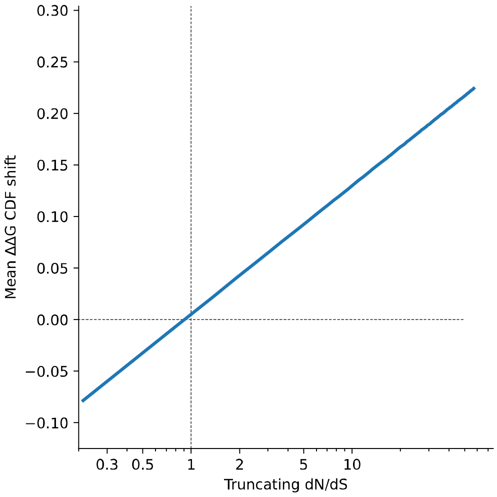

Figure S15 Selection of destabilising missense mutations is correlated with selection of nonsense mutations in mouse oesophagus. Selection of destabilising missense mutations is shown as the shift in the distribution of ΔΔG values between the null model and the observed data. CDF values were used instead of raw ΔΔG values to reduce the influence of extreme outliers (Supplementary Methods). ΔΔG CDF shift is the difference between the mean of the null and observed distributions of ΔΔG CDF values. It is a value between -0.5 (all observed mutations have the minimum ΔΔG possible in the protein structure) and 0.5 (all observed mutations have the maximum ΔΔG possible). A ΔΔG CDF shift of 0 means the mean values of the null and the observed ΔΔG CDF distributions are equal. Homology models of wild type proteins containing at least 50 missense mutations in the mouse oesophagus dataset were used (Supplementary Methods). The average ΔΔG across the homology models was calculated for each residue, and the ΔΔG CDF shift was calculated for each gene. Pearson's correlation coefficient=0.64, two-tailed $p=6e^{-11}$, mean ΔΔG CDF shift vs logarithm of truncating dN/dS ratio. Blue line and shaded region show the linear regression and 95% confidence interval of the regression estimate calculated and plotted using the Python package Seaborn [85].

Supplementary References

1 Martincorena, I. *et al.* Universal Patterns of Selection in Cancer and Somatic Tissues. *Cell* **171**, 1029-1041.e1021 (2017). https://doi.org:https://doi.org/10.1016/j.cell.2017.09.042

2 Mularoni, L., Sabarinathan, R., Deu-Pons, J., Gonzalez-Perez, A. & López-Bigas, N. OncodriveFML: a general framework to identify coding and non-coding regions with cancer driver mutations. *Genome Biology* **17**, 128 (2016). https://doi.org:10.1186/s13059-016-0994-0

3 Brandes, N., Linial, N. & Linial, M. Quantifying gene selection in cancer through protein functional alteration bias. *Nucleic Acids Research* **47**, 6642-6655 (2019). https://doi.org:10.1093/nar/gkz546

4 Gonzalez-Perez, A., Sabarinathan, R. & Lopez-Bigas, N. Local Determinants of the Mutational Landscape of the Human Genome. *Cell* **177**, 101-114 (2019). https://doi.org:https://doi.org/10.1016/j.cell.2019.02.051

5 Goncearenco, A. *et al.* Exploring background mutational processes to decipher cancer genetic heterogeneity. *Nucleic Acids Research* **45**, W514-W522 (2017). https://doi.org:10.1093/nar/gkx367

6 De Strooper, B. *et al.* A presenilin-1-dependent γ-secretase-like protease mediates release of Notch intracellular domain. *Nature* **398**, 518-522 (1999). https://doi.org:10.1038/19083

7 Tagami, S. *et al.* Regulation of Notch Signaling by Dynamic Changes in the Precision of S3 Cleavage of Notch-1. *Molecular and Cellular Biology* **28**, 165 (2008). https://doi.org:10.1128/MCB.00863-07

8 Kopan, R. & Ilagan, M. X. G. The Canonical Notch Signaling Pathway: Unfolding the Activation Mechanism. *Cell* **137**, 216-233 (2009). https://doi.org:10.1016/j.cell.2009.03.045

9 Xu, T.-H. *et al.* Alzheimer's disease-associated mutations increase amyloid precursor protein resistance to γ-secretase cleavage and the Aβ42/Aβ40 ratio. *Cell Discovery* **2**, 16026 (2016). https://doi.org:10.1038/celldisc.2016.26

10 Yang, G. *et al.* Structural basis of Notch recognition by human γ-secretase. *Nature* **565**, 192-197 (2019). https://doi.org:10.1038/s41586-018-0813-8

11 Fay, J. C. & Wu, C.-I. Sequence divergence, functional constraint, and selection in protein evolution. *Annual review of genomics and human genetics* **4**, 213-235 (2003).

12 Luca, V. C. *et al.* Structural basis for Notch1 engagement of Delta-like 4. *Science* **347**, 847 (2015). https://doi.org:10.1126/science.1261093

13 Suckling, R. J. *et al.* Structural and functional dissection of the interplay between lipid and Notch binding by human Notch ligands. *The EMBO Journal* **36**, 2204-2215 (2017). https://doi.org:10.15252/embj.201796632

14 Cooper, G. M. *et al.* Single-nucleotide evolutionary constraint scores highlight disease-causing mutations. *Nat Methods* **7**, 250-251 (2010). https://doi.org:10.1038/nmeth0410-250

15 Wong, C. C. *et al.* Inactivating CUX1 mutations promote tumorigenesis. *Nature Genetics* **46**, 33-38 (2014). https://doi.org:10.1038/ng.2846

16 Fowler, J. C. *et al.* Selection of oncogenic mutant clones in normal human skin varies with body site. *Cancer Discovery* (2020).

17 Sauna, Z. E. & Kimchi-Sarfaty, C. Understanding the contribution of synonymous mutations to human disease. *Nature Reviews Genetics* **12**, 683-691 (2011). https://doi.org:10.1038/nrg3051

18 Kelly, D. F. *et al.* Molecular Structure and Dimeric Organization of the Notch Extracellular Domain as Revealed by Electron Microscopy. *PLOS ONE* **5**, e10532 (2010). https://doi.org:10.1371/journal.pone.0010532

19 Nandagopal, N. *et al.* Dynamic Ligand Discrimination in the Notch Signaling Pathway. *Cell* **172**, 869-880.e819 (2018). https://doi.org:10.1016/j.cell.2018.01.002

20 Willis, A., Jung, E. J., Wakefield, T. & Chen, X. Mutant p53 exerts a dominant negative effect by preventing wild-type p53 from binding to the promoter of its target genes. *Oncogene* **23**, 2330-2338 (2004). https://doi.org:10.1038/sj.onc.1207396

21 Martincorena, I. *et al.* Somatic mutant clones colonize the human esophagus with age. *Science* (2018).

22 Abby, E. *et al.* Notch1 mutation drives clonal expansion in normal esophageal epithelium but impairs tumor growth. *bioRxiv*, 2021.2006.2018.448956 (2021). https://doi.org:10.1101/2021.06.18.448956

23 Huang, C.-H. *et al.* The Structure of a Human p110α/p85α Complex Elucidates the Effects of Oncogenic PI3Kα Mutations. *Science* **318**, 1744 (2007). https://doi.org:10.1126/science.1150799

24 Landrum, M. J. *et al.* ClinVar: improving access to variant interpretations and supporting evidence. *Nucleic acids research* **46**, D1062-D1067 (2018). https://doi.org:10.1093/nar/gkx1153

25 Burke, J. E., Perisic, O., Masson, G. R., Vadas, O. & Williams, R. L. Oncogenic mutations mimic and enhance dynamic events in the natural activation of phosphoinositide 3-kinase p110α (PIK3CA). *Proceedings of the National Academy of Sciences* **109**, 15259 (2012). https://doi.org:10.1073/pnas.1205508109

26 Gkeka, P. *et al.* Investigating the Structure and Dynamics of the PIK3CA Wild-Type and H1047R Oncogenic Mutant. *PLOS Computational Biology* **10**, e1003895 (2014). https://doi.org:10.1371/journal.pcbi.1003895

27 Cordle, J. *et al.* A conserved face of the Jagged/Serrate DSL domain is involved in Notch trans-activation and cis-inhibition. *Nature Structural & Molecular Biology* **15**, 849-857 (2008). https://doi.org:10.1038/nsmb.1457

28 Kitayner, M. *et al.* Structural Basis of DNA Recognition by p53 Tetramers. *Molecular Cell* **22**, 741-753 (2006). https://doi.org:https://doi.org/10.1016/j.molcel.2006.05.015

29 Zhao, Y. *et al.* Crystal Structures of PI3Kα Complexed with PI103 and Its Derivatives: New Directions for Inhibitors Design. *ACS Medicinal Chemistry Letters* **5**, 138-142 (2014). https://doi.org:10.1021/ml400378e

30 Hao, B., Oehlmann, S., Sowa, M. E., Harper, J. W. & Pavletich, N. P. Structure of a Fbw7-Skp1-Cyclin E Complex: Multisite-Phosphorylated Substrate Recognition by SCF Ubiquitin Ligases. *Molecular Cell* **26**, 131-143 (2007). https://doi.org:https://doi.org/10.1016/j.molcel.2007.02.022

31 Dana, J. M. *et al.* SIFTS: updated Structure Integration with Function, Taxonomy and Sequences resource allows 40-fold increase in coverage of structure-based annotations for proteins. *Nucleic Acids Research* **47**, D482-D489 (2018). https://doi.org:10.1093/nar/gky1114

32 Michaud-Agrawal, N., Denning, E. J., Woolf, T. B. & Beckstein, O. MDAnalysis: A toolkit for the analysis of molecular dynamics simulations. *Journal of Computational Chemistry* **32**, 2319-2327 (2011). https://doi.org:10.1002/jcc.21787

33 The UniProt, C. UniProt: a worldwide hub of protein knowledge. *Nucleic Acids Research* **47**, D506-D515 (2018). https://doi.org:10.1093/nar/gky1049

34 Cho, Y., Gorina, S., Jeffrey, P. D. & Pavletich, N. P. Crystal structure of a p53 tumor suppressor-DNA complex: understanding tumorigenic mutations. *Science* **265**, 346 (1994). https://doi.org:10.1126/science.8023157

35 Gorina, S. & Pavletich, N. P. Structure of the p53 Tumor Suppressor Bound to the Ankyrin and SH3 Domains of 53BP2. *Science* **274**, 1001 (1996). https://doi.org:10.1126/science.274.5289.1001

36 Wang, Y., Rosengarth, A. & Luecke, H. Structure of the human p53 core domain in the absence of DNA. *Acta Crystallographica Section D* **63**, 276-281 (2007). https://doi.org:doi:10.1107/S0907444906048499

37 Natan, E. *et al.* Interaction of the p53 DNA-Binding Domain with Its N-Terminal Extension Modulates the Stability of the p53 Tetramer. *Journal of Molecular Biology* **409**, 358-368 (2011). https://doi.org:https://doi.org/10.1016/j.jmb.2011.03.047

38 Arbely, E. *et al.* Acetylation of lysine 120 of p53 endows DNA-binding specificity at effective physiological salt concentration. *Proceedings of the National Academy of Sciences* **108**, 8251 (2011). https://doi.org:10.1073/pnas.1105028108

39 Kitayner, M. *et al.* Diversity in DNA recognition by p53 revealed by crystal structures with Hoogsteen base pairs. *Nature Structural & Molecular Biology* **17**, 423-429 (2010). https://doi.org:10.1038/nsmb.1800

40 Chen, Y., Dey, R. & Chen, L. Crystal Structure of the p53 Core Domain Bound to a Full Consensus Site as a Self-Assembled Tetramer. *Structure* **18**, 246-256 (2010). https://doi.org:https://doi.org/10.1016/j.str.2009.11.011

41 Chen, Y. *et al.* Structure of p53 binding to the BAX response element reveals DNA unwinding and compression to accommodate base-pair insertion. *Nucleic Acids Research* **41**, 8368-8376 (2013). https://doi.org:10.1093/nar/gkt584

42 Bethuyne, J. *et al.* A nanobody modulates the p53 transcriptional program without perturbing its functional architecture. *Nucleic Acids Research* **42**, 12928-12938 (2014). https://doi.org:10.1093/nar/gku962

43 Martinez-Zapien, D. *et al.* Structure of the E6/E6AP/p53 complex required for HPV-mediated degradation of p53. *Nature* **529**, 541-545 (2016). https://doi.org:10.1038/nature16481

44 Vainer, R., Cohen, S., Shahar, A., Zarivach, R. & Arbely, E. Structural Basis for p53 Lys120-Acetylation-Dependent DNA-Binding Mode. *Journal of Molecular Biology* **428**, 3013-3025 (2016). https://doi.org:https://doi.org/10.1016/j.jmb.2016.06.009

45 Golovenko, D. *et al.* New Insights into the Role of DNA Shape on Its Recognition by p53 Proteins. *Structure* **26**, 1237-1250.e1236 (2018). https://doi.org:https://doi.org/10.1016/j.str.2018.06.006

46 Ehebauer, Matthias T., Chirgadze, Dimitri Y., Hayward, P., Martinez Arias, A. & Blundell, Tom L. High-resolution crystal structure of the human Notch 1 ankyrin domain. *Biochemical Journal* **392**, 13-20 (2005). https://doi.org:10.1042/bj20050515

47 Nam, Y., Sliz, P., Song, L., Aster, J. C. & Blacklow, S. C. Structural Basis for Cooperativity in Recruitment of MAML Coactivators to Notch Transcription Complexes. *Cell* **124**, 973-983 (2006). https://doi.org:https://doi.org/10.1016/j.cell.2005.12.037

48 Gordon, W. R. *et al.* Structure of the Notch1-negative regulatory region: implications for normal activation and pathogenic signaling in T-ALL. *Blood* **113**, 4381-4390 (2009). https://doi.org:10.1182/blood-2008-08-174748

49 Wu, Y. *et al.* Therapeutic antibody targeting of individual Notch receptors. *Nature* **464**, 1052-1057 (2010). https://doi.org:10.1038/nature08878

50 Taylor, P. *et al.* Fringe-mediated extension of O-linked fucose in the ligand-binding region of Notch1 increases binding to mammalian Notch ligands. *Proceedings of the National Academy of Sciences* **111**, 7290 (2014). https://doi.org:10.1073/pnas.1319683111

51 Weisshuhn, Philip C. *et al.* Non-Linear and Flexible Regions of the Human Notch1 Extracellular Domain Revealed by High-Resolution Structural Studies. *Structure* **24**, 555-566 (2016). https://doi.org:10.1016/j.str.2016.02.010

52 Li, Z. *et al.* Structural basis of Notch O-glucosylation and O–xylosylation by mammalian protein–O-glucosyltransferase 1 (POGLUT1). *Nature Communications* **8**, 185 (2017). https://doi.org:10.1038/s41467-017-00255-7

53 Hackos, David H. *et al.* Positive Allosteric Modulators of GluN2A-Containing NMDARs with Distinct Modes of Action and Impacts on Circuit Function. *Neuron* **89**, 983-999 (2016). https://doi.org:https://doi.org/10.1016/j.neuron.2016.01.016

54 Volgraf, M. *et al.* Discovery of GluN2A-Selective NMDA Receptor Positive Allosteric Modulators (PAMs): Tuning Deactivation Kinetics via Structure-Based Design. *Journal*

of Medicinal Chemistry **59**, 2760-2779 (2016). https://doi.org:10.1021/acs.jmedchem.5b02010
55 Villemure, E. *et al.* GluN2A-Selective Pyridopyrimidinone Series of NMDAR Positive Allosteric Modulators with an Improved in Vivo Profile. *ACS Medicinal Chemistry Letters* **8**, 84-89 (2017). https://doi.org:10.1021/acsmedchemlett.6b00388
56 Heffron, T. P. *et al.* The Rational Design of Selective Benzoxazepin Inhibitors of the α-Isoform of Phosphoinositide 3-Kinase Culminating in the Identification of (S)-2-((2-(1-Isopropyl-1H-1,2,4-triazol-5-yl)-5,6-dihydrobenzo[f]imidazo[1,2-d][1,4]oxazepin-9-yl)oxy)propanamide (GDC-0326). *Journal of Medicinal Chemistry* **59**, 985-1002 (2016). https://doi.org:10.1021/acs.jmedchem.5b01483
57 Qin, L.-Y. *et al.* Discovery of 7-(3-(piperazin-1-yl)phenyl)pyrrolo[2,1-f][1,2,4]triazin-4-amine derivatives as highly potent and selective PI3Kδ inhibitors. *Bioorganic & Medicinal Chemistry Letters* **27**, 855-861 (2017). https://doi.org:https://doi.org/10.1016/j.bmcl.2017.01.016
58 Song, K., Yang, X., Zhao, Y., Jian, Z. *Crystal structure of PI3K complex with an inhibitor, doi:10.2210/pdb5XGI/pdb* (2018).
59 Ouvry, G. *et al.* Impact of Minor Structural Modifications on Properties of a Series of mTOR Inhibitors. *ACS Medicinal Chemistry Letters* **10**, 1561-1567 (2019). https://doi.org:10.1021/acsmedchemlett.9b00401
60 Fradera, X. *et al.* Design of selective PI3Kδ inhibitors using an iterative scaffold-hopping workflow. *Bioorganic & Medicinal Chemistry Letters* **29**, 2575-2580 (2019). https://doi.org:https://doi.org/10.1016/j.bmcl.2019.08.004
61 Wood, E. R. *et al.* 6-Ethynylthieno[3,2-d]- and 6-ethynylthieno[2,3-d]pyrimidin-4-anilines as tunable covalent modifiers of ErbB kinases. *Proceedings of the National Academy of Sciences* **105**, 2773 (2008). https://doi.org:10.1073/pnas.0708281105
62 Qiu, C. *et al.* Mechanism of Activation and Inhibition of the HER4/ErbB4 Kinase. *Structure* **16**, 460-467 (2008). https://doi.org:https://doi.org/10.1016/j.str.2007.12.016
63 Liu, P., Bouyain, S., Eigenbrot, C. & Leahy, D. J. The ErbB4 extracellular region retains a tethered-like conformation in the absence of the tether. *Protein Science* **21**, 152-155 (2012). https://doi.org:https://doi.org/10.1002/pro.753
64 Yalla, K. *et al.* FBXW7 regulates DISC1 stability via the ubiquitin-proteosome system. *Molecular Psychiatry* **23**, 1278-1286 (2018). https://doi.org:10.1038/mp.2017.138
65 Cho, H.-S. *et al.* Structure of the extracellular region of HER2 alone and in complex with the Herceptin Fab. *Nature* **421**, 756-760 (2003). https://doi.org:10.1038/nature01392
66 Garrett, T. P. J. *et al.* The Crystal Structure of a Truncated ErbB2 Ectodomain Reveals an Active Conformation, Poised to Interact with Other ErbB Receptors. *Molecular Cell* **11**, 495-505 (2003). https://doi.org:https://doi.org/10.1016/S1097-2765(03)00048-0
67 Huyvetter, M. *et al.* ¹³¹I-labeled Anti-HER2 Camelid sdAb as a Theranostic Tool in Cancer Treatment. *Clinical Cancer Research* **23**, 6616 (2017). https://doi.org:10.1158/1078-0432.CCR-17-0310
68 Garrett, T. P. J. *et al.* Crystal Structure of a Truncated Epidermal Growth Factor Receptor Extracellular Domain Bound to Transforming Growth Factor α. *Cell* **110**, 763-773 (2002). https://doi.org:https://doi.org/10.1016/S0092-8674(02)00940-6

69 Li, S. *et al.* Structural basis for inhibition of the epidermal growth factor receptor by cetuximab. *Cancer Cell* **7**, 301-311 (2005). https://doi.org:https://doi.org/10.1016/j.ccr.2005.03.003
70 Lim, Y. *et al.* GC1118, an Anti-EGFR Antibody with a Distinct Binding Epitope and Superior Inhibitory Activity against High-Affinity EGFR Ligands. *Molecular Cancer Therapeutics* **15**, 251 (2016). https://doi.org:10.1158/1535-7163.MCT-15-0679
71 Chen, C., Gorlatova, N., Kelman, Z. & Herzberg, O. Structures of p63 DNA binding domain in complexes with half-site and with spacer-containing full response elements. *Proceedings of the National Academy of Sciences* **108**, 6456 (2011). https://doi.org:10.1073/pnas.1013657108
72 Chen, C., Gorlatova, N. & Herzberg, O. Pliable DNA Conformation of Response Elements Bound to Transcription Factor p63*. *Journal of Biological Chemistry* **287**, 7477-7486 (2012). https://doi.org:https://doi.org/10.1074/jbc.M111.315820
73 Xu, X. *et al.* Insights into Autoregulation of Notch3 from Structural and Functional Studies of Its Negative Regulatory Region. *Structure* **23**, 1227-1235 (2015). https://doi.org:https://doi.org/10.1016/j.str.2015.05.001
74 Bernasconi-Elias, P. *et al.* Characterization of activating mutations of NOTCH3 in T-cell acute lymphoblastic leukemia and anti-leukemic activity of NOTCH3 inhibitory antibodies. *Oncogene* **35**, 6077-6086 (2016). https://doi.org:10.1038/onc.2016.133
75 Himanen, J. P. *et al.* Architecture of Eph receptor clusters. *Proceedings of the National Academy of Sciences* **107**, 10860 (2010). https://doi.org:10.1073/pnas.1004148107
76 Araç, D. *et al.* A novel evolutionarily conserved domain of cell-adhesion GPCRs mediates autoproteolysis. *The EMBO Journal* **31**, 1364-1378 (2012). https://doi.org:https://doi.org/10.1038/emboj.2012.26
77 Barr, A. J. *et al.* Large-Scale Structural Analysis of the Classical Human Protein Tyrosine Phosphatome. *Cell* **136**, 352-363 (2009). https://doi.org:https://doi.org/10.1016/j.cell.2008.11.038
78 Wernimont, A. K., Dong, A., Seitova, A., Crombet, L., Khutoreskaya, G., Edwards, A.M., Arrowsmith, C.H., Bountra, C., Weigelt, J., Cossar, D., Dobrovetsky, E. *Crystal Structure of Metabotropic glutamate receptor 3 precursor in presence of LY341495 antagonist, doi: 10.2210/pdb3SM9/pdb* (2011).
79 Mitternacht, S. FreeSASA: An open source C library for solvent accessible surface area calculations. *F1000Research* **5** (2016).
80 Phipson, B. & Smyth Gordon, K. in *Statistical Applications in Genetics and Molecular Biology* Vol. 9 (2010).
81 Smith, Z. R. & Wells, C. S. in *annual meeting of the Northeastern Educational Research Association, Kerhonkson, New York.*
82 Virtanen, P. *et al.* SciPy 1.0: fundamental algorithms for scientific computing in Python. *Nat Methods* (2020). https://doi.org:10.1038/s41592-019-0686-2
83 Luca, V. C. *et al.* Notch-Jagged complex structure implicates a catch bond in tuning ligand sensitivity. *Science* **355**, 1320 (2017). https://doi.org:10.1126/science.aaf9739
84 Benjamini, Y. & Hochberg, Y. Controlling the false discovery rate: a practical and powerful approach to multiple testing. *Journal of the royal statistical society. Series B (Methodological)*, 289-300 (1995).
85 Waskom, M. L. Seaborn: statistical data visualization. *Journal of Open Source Software* **6**, 3021 (2021).
